# Differentiation of human induced pluripotent stem cells into retinal pigment epithelium cells during culture on peptide-grafted hydrogels

**DOI:** 10.1093/rb/rbaf035

**Published:** 2025-04-26

**Authors:** Jun Liu, Qian Liu, Minmei Guo, Chengyu Jiang, Jianyang Chen, Ting Wang, Tzu-Cheng Sung, Shih-Jie Chou, Shih-Hwa Chiou, Guoping Fan, Akon Higuchi

**Affiliations:** State Key Laboratory of Eye Health, Eye Hospital, Wenzhou Medical University, Wenzhou, Zhejiang 325027, China; State Key Laboratory of Eye Health, Eye Hospital, Wenzhou Medical University, Wenzhou, Zhejiang 325027, China; State Key Laboratory of Eye Health, Eye Hospital, Wenzhou Medical University, Wenzhou, Zhejiang 325027, China; State Key Laboratory of Eye Health, Eye Hospital, Wenzhou Medical University, Wenzhou, Zhejiang 325027, China; State Key Laboratory of Eye Health, Eye Hospital, Wenzhou Medical University, Wenzhou, Zhejiang 325027, China; State Key Laboratory of Eye Health, Eye Hospital, Wenzhou Medical University, Wenzhou, Zhejiang 325027, China; State Key Laboratory of Eye Health, Eye Hospital, Wenzhou Medical University, Wenzhou, Zhejiang 325027, China; Department of Medical Research, Taipei Veterans General Hospital, Taipei 112201, Taiwan, China; Institute of Pharmacology, School of Medicine, National Yang Ming Chiao Tung University, Taipei 112304, Taiwan, China; Department of Medical Research, Taipei Veterans General Hospital, Taipei 112201, Taiwan, China; Institute of Pharmacology, School of Medicine, National Yang Ming Chiao Tung University, Taipei 112304, Taiwan, China; Department of Ophthalmology, Taipei Veterans General Hospital, Taipei 11217, Taiwan, China; Department of Human Genetics, David Geffen School of Medicine, UCLA, Los Angeles, CA 90095, USA; State Key Laboratory of Eye Health, Eye Hospital, Wenzhou Medical University, Wenzhou, Zhejiang 325027, China; Department of Chemical and Materials Engineering, National Central University, Taoyuan 32001, Taiwan, China; R&D Center for Membrane Technology, Chung Yuan Christian University, Taoyuan 320, Taiwan, China

**Keywords:** peptide, hydrogel, human pluripotent stem cell, retinal pigment epithelium, subretinal transplantation

## Abstract

A variety of novel peptide-grafted hydrogels, of which peptides were derived from vitronectin (PQVTRGDVFTMP) or the laminin β4 chain (PMQKMRGDVFSP), were prepared in this study. The peptide-grafted hydrogels promoted the adhesion, proliferation and colony formation of hiPSCs and maintained their pluripotency up to passage 5 under xeno-free conditions. We successfully generated RPE cells from hiPSCs using one of the most suitable xeno-free peptide-grafted hydrogels, KVN2CK (KGCGGKGG-PQVTRGDVFTMP), which was derived from vitronectin, and confirmed the effect of these hiPSC-derived RPE cells in a rat retinal degeneration model (Royal College of Surgeons (RCS) rats) via subretinal transplantation, when we investigated functional improvements in vision in RCS rats after the transplantation of hiPSC-derived RPE cells. Our novel peptide-grafted hydrogels provided a safe and robust platform for generating single-layer hiPSC-derived RPE cells under xeno-free conditions, which indicates the potential of these hydrogels for stem cell therapy for retinal degenerative diseases in the future.

## Graphical abstract

**Figure:**
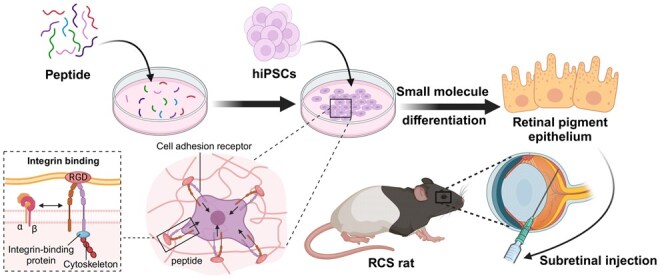

## Introduction

Retinal pigment epithelium (RPE) cells are a hexagonal monolayer of flat cells that play crucial roles in maintaining the homeostasis and function of the retina [1]. The deterioration of RPE function is a major factor contributing to various genetic and nongenetic retinal degenerative diseases, such as Stargardt's disease (SD) and age-related macular degeneration (AMD) [2, 3]. AMD is a progressive and vision-threatening disease that affects mainly elderly people and is a leading cause of irreversible blindness in Western countries. The risk of early AMD in individuals over 75 years old is 25%, whereas the risk of late-stage AMD is 8%, with the number of cases expected to increase due to the aging population [4].

Currently, patients with wet AMD can be treated with monthly intravitreal injections of anti-vascular endothelial growth factor (anti-VEGF) drugs [4, 5]. Although anti-VEGF therapy can delay the progression of wet AMD in some patients, prolonged use of these drugs is expensive and may impact the viability of RPE cells and affect RPE function [6, 7]. However, human pluripotent stem cell (hPSC)-based therapy using human embryonic stem cell (hESC)-derived or human induced pluripotent stem cell (hiPSC)-derived RPE cell suspensions or RPE cell patches for the treatment of both wet and dry AMD patients is in clinical trials and has demonstrated potential efficacy [8–18].

hPSCs are cultured primarily on surfaces coated with Matrigel, recombinant laminin or vitronectin protein [19–22]. Matrigel is widely used in hPSC culture, but it is derived from mouse Engelbreth–Holm–Swarm sarcoma [23, 24]. The complex, ambiguous and variable composition of Matrigel limits its applicability in basic research, therapeutic cell manufacturing and cell-based analysis [25]. Moreover, the inconsistent biochemical properties across batches of Matrigel reduces reproducibility in cell experiments [26, 27]. Significant progress has been made in developing xeno-free and chemically defined materials for hPSC culture and adhesion. Matrigel is typically replaced with human recombinant proteins, such as human recombinant laminin [28–30] and human recombinant vitronectin [22, 31, 32], to support hPSC proliferation.

There has been an increasing focus on the use of peptides to increase the bioactivity of synthetic materials for hPSC culture in recent years [33–35]. The application of synthetic peptides has overcome the limitations of natural or full-length proteins, such as their large size, instability and conformational changes due to variations in temperature and pH [36]. Synthetic peptide-grafted biomaterials can provide adhesive sites for hPSCs with high reproducibility. Lawley *et al.* developed novel synthetic peptides to support hPSC culture, named Synthemax-R and Synthemax II-SC; these peptides are animal component-free copolymers containing the RGD (Arg-Gly-Asp) sequence [37, 38]. These copolymers contain bioactive peptides extracted from human extracellular matrix (ECM) proteins (vitronectin) and are covalently bound to acrylate molecules, which can be coated on tissue culture polystyrene (TCP) plates [37, 38].

In our previous studies [39–41], we designed several peptide-grafted polyvinyl alcohol-itaconic acids (PAIs), which effectively promoted the proliferation of hPSCs. The differentiation of hPSCs cultured on peptide-grafted PAI hydrogels has been studied only for cells of the mesoderm lineage: mesenchymal stem cells [42] and cardiomyocytes [43, 44]. However, hPSC differentiation into cells of the ectoderm lineage, namely, RPE cells, on peptide-grafted PAI hydrogels has not yet been investigated, as most protocols for the differentiation of hPSCs into RPE cells use Matrigel-coated dishes [45, 46] or ECM proteins [47–51].

In this study, we designed and developed novel PAI hydrogels, which were conjugated with several specifically designed peptides to maintain hPSC proliferation and support their differentiation into RPE cells. Specifically, we investigated the effects of several peptides derived from vitronectin (PQVTRGDVFTMP) and laminin β4 (PMQKMRGDVFSP), which may promote the adhesion and proliferation of hPSCs and hPSC-derived RPE cells through αvβ5 [37, 40, 44, 52–54] or α6β1 [44, 54] integrin, respectively. hiPSC proliferation and differentiation into RPE cells on peptide-grafted PAI hydrogels in xeno-free culture conditions were compared with those cultured on Matrigel-coated TCP dishes in xeno-containing culture conditions in this study. Moreover, we investigated functional improvements in vision in a rat retinal degeneration model after the transplantation of hPSC-derived RPE cells, which were prepared on xeno-free cell culture biomaterials containing specific peptide-grafted PAI hydrogels.

## Materials and methods

### Preparation of the surface of the peptide-grafted PAI hydrogels

To prepare a transparent poly(vinyl alcohol-co-itaconic acid) (PAI, 98 mol% hydrolyzed with 1.3 mol% itaconic acid), 2 ml 0.05 wt% PAI solution was added to each well of a 6-well TCP dish and then dried to gain the PAI film. The PAI film was then crosslinked by immersion in 1 ml aqueous solution containing Na_2_SO_4_ (20 w/v%) (JT-3375-01, Sigma-Aldrich, USA), H_2_SO_4_ (1 w/v%) (339741, Sigma-Aldrich, USA) and glutaraldehyde (0.1 w/v%) (G6257, Sigma-Aldrich, USA). Our previous studies suggested that an optimal crosslinking reaction time of 24 h promotes the proliferation and induction of hPSCs on PAI hydrogels grafted with peptides [39, 40]. The crosslinking solution was replaced with deionized water after crosslinking, and the hydrogel surface was washed three times for 5 min each. The PAI-coated 6-well plate was immersed in 75 v/v% ethanol overnight for sterilization.

**Figure 1.**
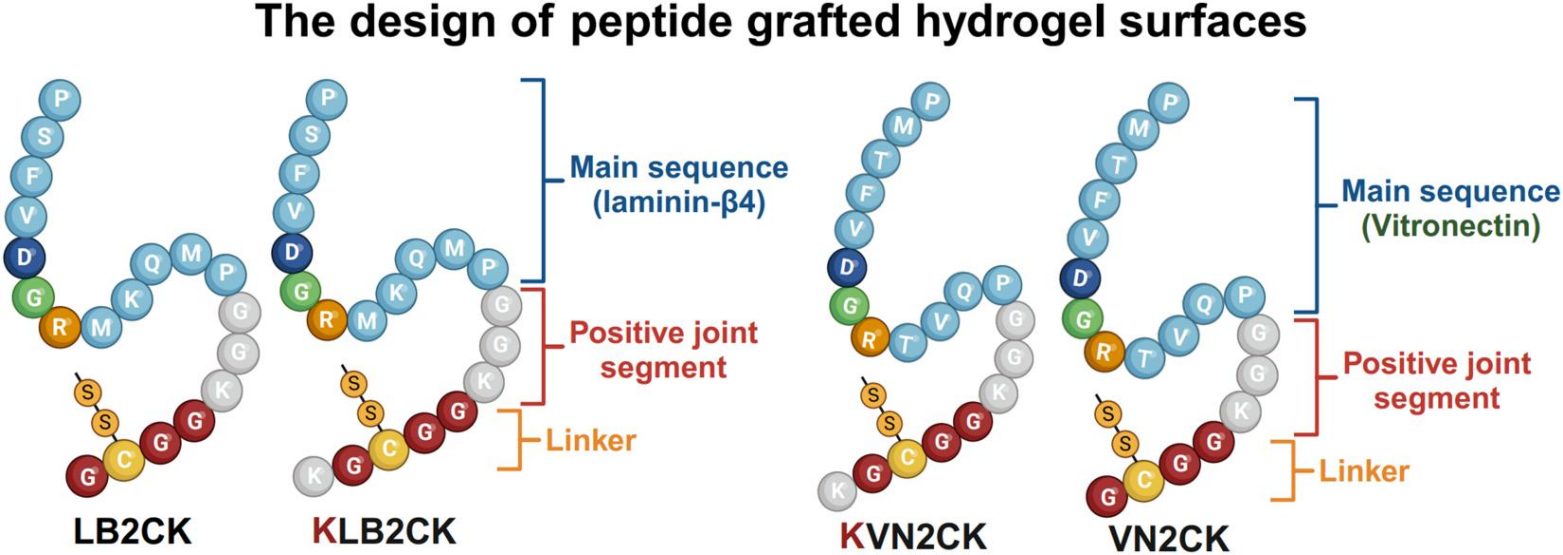
The design of peptide-grafted hydrogel surfaces. Schematic design of the peptide sequences derived from the laminin-β4 chain (PMQKMRGDVFSP) and the vitronectin protein (PQVTRGDVFTMP). K: lysine, G: glycine, C: cysteine.

The PAI hydrogels were reacted with EDC (1-ethyl-3-(-3-dimethylaminopropyl)carbodiimide hydrochloride) (E7750, Sigma-Aldrich, USA) and NHS (N-hydroxysuccinimide) (56480, Sigma-Aldrich, USA) using EDC/NHS chemistry [55] to conjugate a monopeptide (VN2CK, KVN2CK, LB2CK, KLB2CK) or a dipeptide (KVN2CK + KLB2CK, VN2CK + LB2CK), with the peptides VN2CK and KVN2CK derived from the vitronectin sequence for cell adhesion (PQVTRGDVFTMP) and the peptides LB2CK and KLB2CK derived from the laminin β4 chain (PMQKMRGDVFSP). The peptide sequences of VN2CK, KVN2CK, LB2CK and KLB2CK were GCGGKGG-PQVTRGDVFTMP, KGCGGKGG-PQVTRGDVFTMP, GCGGKGG-PMQKMRGDVFSP and KGCGGKGG-PMQKMRGDVFSP, respectively and are shown in Figure 1.

The peptides were dissolved in pure water at pH 7.4 and diluted to the desired concentration (50, 100, 200, 250, 500, 750, 1000 or 1500 μg/ml) before conjugation to the PAI hydrogels. The peptide mixture was prepared by mixing the two peptides at an equal mass ratio. A crosslinking solution containing 10 mg/ml EDC and 10 mg/ml NHS was used to activate the PAI hydrogel at 4°C for 6 h, and the hydrogel was then immersed in peptide solutions of varying concentrations to bind the peptides to the PAI hydrogels. Any remaining peptides were removed by soaking in pure water for 12 h before storage at 4°C until the time of cell culture. The peptides were obtained from Genscript Biotech Corporation (Zhenjiang, Jiangsu, China).

### Surface characterization of peptide-grafted PAI hydrogels

The chemical composition of the surface of the peptide-grafted PAI hydrogels was analysed via X-ray photoelectron spectroscopy (XPS) (ESCALAB 250Xi, Thermo Scientific, USA). The XPS system was calibrated by referencing the C1s peak at 285 eV. The hydrophilicity and hydrophobicity of the surfaces were evaluated via contact angle measurements at room temperature using sessile drop method by Theta Flex instrument (Biolin Scientific, Gothenburg, Sweden). Three samples of each peptide-grafted PAI hydrogel or PAI hydrogel were analysed to calculate the average water contact angle and standard deviation to ensure the accuracy of the data.

### hiPSC culture

We used two hiPSC lines. One cell line is hiPSC (HPS0077) obtained from RIKEN BioResource Center (Tsukuba, Japan). The other hiPSC line is MIX2 generated in our laboratory in a previous study [56].

The hiPSCs were cultivated on Matrigel-coated plates with chemically defined mTeSR1 medium (85850, StemCell Technologies, Canada) in a 37°C humidified 5% CO_2_ incubator (Thermo Fisher, USA). Once the cell confluency reached 80–90%, the hiPSCs were detached using Accutase (A6964, Sigma, USA) at 37°C for 3–5 min. The hiPSCs were then seeded on Matrigel-coated or peptide-grafted PAI hydrogel surfaces at a density of 2.5 × 10^4^ cells/cm^2^. The medium was changed daily.

### Analysis of the differentiation ability of embryoid bodies

The hiPSCs cultured on the surface of the KVN2CK-grafted hydrogels were detached using 500 unit/ml of Accutase solution (3–5 min treatment at 37°C), and the cell pellets were suspended on ultralow attachment microplates (3471, Corning, USA) with 2.0 × 10^5^ cells/ml concentration in Essential 6™ medium (A1516401, Gibco, USA) for 14 days. After 14 days of suspension culture, embryoid bodies (EBs) were formed with the size around 200–400 μm. Subsequently, the EBs were transferred to Matrigel-coated Petri dishes with Essential 6™ medium for EB attachment and cell expansion of 6 days for analysis of the cells derived from three germ layers using immunostaining method. The Essential 6™ medium was replaced every two days until the EBs were attached to and spread on the Matrigel-coated surface. Immunohistochemical analysis was performed using antibodies against α-smooth muscle actin (α-SMA; mesoderm), alpha-fetoprotein (AFP; endoderm) and glial fibrillary acidic protein (GFAP; ectoderm).

### Differentiation of hiPSCs into RPE cells

The differentiation of hiPSCs into RPE cells was conducted in accordance with the protocol proposed by Maruotti *et al.* [45] and Smith *et al.* [46], with minor modifications. Initially, hiPSCs reached 80–90% confluence. The culture medium was subsequently changed to RPE differentiation medium (DM). This DM comprises 85% DMEM/F12 (10565, Thermo Fisher Scientific, USA) and 15% KnockOut Serum Replacement (KSR) (10828, Thermo Fisher Scientific, USA), supplemented with 1% MEM nonessential amino acids (NEAAs) (11140, Thermo Fisher Scientific, USA), 1% Anti-Anti (15240062, Thermo Fisher Scientific, USA) and 0.1 mM β-mercaptoethanol (21985023, Thermo Fisher Scientific, USA). From D2 to D14, the DM was supplemented with 10 μM nicotinamide (NIC) (N3376, Sigma-Aldrich, USA) and 20 nM chetomin (CTM) (C9623, Sigma-Aldrich, USA). After D14, the NIC concentration was adjusted to 10 μM, and the medium was replaced daily with DM for an additional 2 weeks. On D28, the culture medium was changed to RPE medium, which was composed of 70% DMEM (10566, Thermo Fisher Scientific, USA) and 30% F12 (11765, Thermo Fisher Scientific, USA) supplemented with 2% B27 and 1% Anti-Anti, until pigment cells became visible. Approximately 6 weeks post-differentiation, discernible pigment cell clusters were detected. These clusters were manually isolated and transferred to Matrigel-coated TCP plates or peptide-grafted PAI hydrogel surfaces for monolayer culture and subsequent expansion.

### Immunofluorescence analysis of the cells

Following a 15-min in 4% paraformaldehyde (PFA) solution, cells in the plates were permeabilized for 10–15 min in 0.25% Triton X-100 in phosphate-buffered saline (PBS). This was followed by a 60-min incubation period with immunostaining blocking buffer (P0102, Beyotime, China). The cells were incubated overnight at 4°C with the following primary antibodies: anti-OCT4 antibody (PA5-27438, Thermo Fisher Scientific, USA, 1:200), anti-SOX2 antibody (48-1400, Thermo Fisher Scientific, USA, 1:100), anti-SSEA4 antibody (MA1-021, Thermo Fisher Scientific, USA, 1:100), anti-NANOG antibody (MA1-017, Thermo Fisher Scientific, USA, 1:100), anti-α-SMA antibody (14-9760, Thermo Fisher Scientific, USA, 1:500), anti-AFP antibody (PA5-21004, Thermo Fisher Scientific, USA, 1:500), anti-GFAP antibody (ab207165, Abcam, UK), anti-ZO-1 antibody (40-2200, Thermo Fisher Scientific, USA, 1:200), anti-PAX6 antibody (13B10-1A10, Thermo Fisher Scientific, USA, 1:200), anti-MITF antibody (ab3201, Abcam, UK, 1:500) and anti-RPE65 antibody (MA5-32633, Invitrogen, 1:200). The cells were exposed to the following secondary antibodies at a dilution of 1:1000 for 1 h at room temperature: goat anti-rabbit IgG H&L (Alexa Fluor^®^ 488, ab150077, Abcam, UK), goat anti-rabbit IgG H&L (Alexa Fluor^®^ 555, ab150078, Abcam, UK), goat anti-mouse IgG H&L (Alexa Fluor^®^ 488, ab150113, Abcam, UK) and goat anti-mouse IgG H&L (Alexa Fluor^®^ 555, ab150114, Abcam, UK). The cells were subsequently stained with 4′,6-diamidino-2-phenylindole (DAPI) (C1006, Beyotime, China) for 5 min. The cells were washed with immunofluorescence washing solution three times before each step. Immunofluorescence images were captured using with a fluorescence microscope (Eclipse Ti-U, Nikon Instruments, Tokyo, Japan) or a confocal microscope (LSM900, Zeiss, Germany).

### Flow cytometry analysis of the cells

hiPSCs and hiPSC-derived RPE cells cultured on KVN2CK-grafted hydrogels and Matrigel-coated TCP plate surfaces were dissociated into single cells at 37°C using Accutase. The cells were subsequently treated with 4% PFA solution for 20 min, followed by treatment with 90% methanol solution for 15 min. The cells were then incubated at 37°C for 30 min with a primary anti-RPE65 antibody (MA5-32633, Invitrogen, 1:200) anti-OCT4 antibody (PA5-27438, Invitrogen, 1:100), anti-SOX2 antibody (48-1400, Invitrogen, 1:100), anti-NANOG antibody (MA1-017, Invitrogen, 1:100) or anti-SSEA4 antibody (MA1-021, Invitrogen, 1:100) and secondary antibodies, goat anti-rabbit IgG H&L (PE) (ab72465, Abcam, UK, 1:500) or goat anti-mouse IgG H&L (FITC) (ab6785, Abcam, 1:500). A rabbit IgG isotype control (10500C, Invitrogen, USA) and a mouse IgG isotype control (MA5-14453, Invitrogen, USA) were also used. The cells were washed twice with fluorescence-activated cell sorting (FACS) buffer (PBS, 2% fetal bovine serum (FBS) and 0.1% NaN_3_ sodium azide) after each step of the procedure. In the final step, the cells were resuspended in FACS buffer and immediately analysed using a flow cytometer (C6 Plus, Becton Dickinson, USA), and the data were analysed using FlowJo software (Becton Dickinson, USA).

### Karyotype analysis

Karyotype analysis of RPE cells derived from hiPSCs (HPS0077 or MIX2) was performed via G-banding (1000× microscope image magnification; 350–400 band-count resolution) by experts at the Second Affiliated Hospital of Wenzhou Medical University.

### ELISA for trophic factors secreted by hiPSC-derived RPE cells

The cultivation media were obtained from hiPSC (HPS0077)-derived RPE cells, which were cultivated on KVN2CK-grafted PAI hydrogel surface and Matrigel-coated dishes at two days after the media change. Then, the media were centrifuged at 1000×g for 18 min to get debris-free supernatant and stored at −20°C until their usage. Furthermore, cell numbers were calculated and recorded for subsequent calculations. The secreted amount of Vascular Endothelial Growth Factor (VEGF) and Pigment Epithelium Derived Factor (PEDF) was evaluated with VEGF-ELISA kit (JL18341, Jianglai Bio, Shanghai, China) and PEDF-ELISA kit (JL10799, Jianglaibio, Shanghai, China), respectively, according to the manufacturer’s instructions. Standard protein solution having gradient concentration in the kit was utilized to obtain standard curve for estimating the concentration of each trophic factor. The secretions of trophic factors of each group of cells were normalized by reducing the corresponded control (cell culture media only). A total of three independent cell solutions were collected from each cell condition for evaluating and statistical analysis.

### Animal experiments

RCS rats were used in this study, and they were bred in the specific pathogen-free (SPF)-grade animal facility at Wenzhou Medical University. RCS rats were anesthetized after intraperitoneal injection of 1% sodium pentobarbital (30 mg/kg). In addition, topical application of 0.4% oxybuprocaine hydrochloride (Alcaine, Alconwas, USA) was applied as needed to achieve corneal anesthesia. The immunosuppressive agent cyclosporine A (C106893, Aladdin, Shanghai, China) was administered in the drinking water at a concentration of 210 mg/l following transplantation, and this regimen was sustained until the rats were euthanized. The animal experiments were approved by the Laboratory Animal Ethics Committee of Wenzhou Medical University (wydw2022-0474).

### Transplantation of hiPSC-derived RPE cells into RCS rats

hiPSC-derived RPE cells were isolated, quantified and subsequently stained with CellTracker™ CM-DiI (C2925, Thermo Fisher Scientific, USA) following treatment with Accutase. The cells were resuspended in PBS at a concentration of 5 × 10^4^ cells/μl. Twenty-one-day-old RCS rats received an injection of 1 × 10^5^ hiPSC-derived RPE cells (2 μl of 5 × 10^4^ cells/μl) or PBS (2 μl, negative control) into the subretinal space of right eyes. RCS rats were randomly assigned to multiple transplanted groups (transplantation of (a) PBS (negative control), (b) hiPSC (HPS0077)-derived RPE cells cultured on Matrigel-coated TCP dishes, (c) hiPSC (MIX2)-derived RPE cells cultured on Matrigel-coated TCP dishes, (d) hiPSC (HPS0077)-derived RPE cells cultured on KVN2CK-grafted PAI hydrogels, (e) hiPSC (MIX2)-derived RPE cells cultured on KVN2CK-grafted PAI hydrogels) with five RCS rats in each group (totally 25 RCS rats). Images of the fundus were captured promptly using a retinal imaging system for small animals (Phoenix Research Labs, Pleasonton, CA) to visualize the fluorescence and morphology of the fundus, thereby ensuring successful transplantation.

### Optomotor response evaluation

The optomotor response (OMR) was used to assess the visual function of RCS rats. The OMR system (PhenoSys, Berlin, Germany) is composed of a box with four screens positioned around a circular platform at the center of the box, where the RCS rats were placed [57, 58]. The rats were exposed to visual stimuli on the screens. The collected data were subsequently processed with a software program to generate visual thresholds (OMR scores). We designed and implemented a virtual stimulation protocol involving spatial frequencies of 0.05, 0.1, 0.15, 0.2, 0.3, 0.4 and 0.5 cycles per degree (c/deg). Each spatial frequency was randomly presented for 60 s at a speed of 12°/s. The OMR score represents the ratio of consistency to inconsistency between the body/head movement of the animal and the moving patterns on the surrounding screens.

### Electroretinography evaluation

Electroretinography (ERG) (RETI-Port21, Roland, Germany) was used to record the electrical responses of retinal cells in RCS rats at 4 and 8 weeks to evaluate the visual function of RCS rats [15, 57]. The rats were placed in a dark room overnight for dark adaptation before the ERG test. The rats were maintained at a constant temperature of 37°C throughout the ERG testing process. The pupils of the rats were dilated with tropicamide eye drops, followed by eye surface anesthesia with proparacaine eye drops (Alcaine, Alcon, USA) and treatment with ofloxacin eye ointment (Dikeluo, Sinqi Pharmaceutical, China) to prevent eye dryness and bacterial infection. Two gold ring electrodes (positive) were placed on the cornea of each eye. Two needle reference electrodes (negative) were inserted under the skin of the cheeks, and a ground electrode was inserted under the skin of the tail. The dark-adapted electroretinograms were then recorded sequentially at stimulus light intensities of 0.01, 3.0 and 10.0 cd/m^2^. Three trial responses of each ERG experiment were averaged to create a standard waveform.

### Histological analysis

Eyeballs were collected at 4 and 8 weeks post-transplantation after the ERG test. The eyeballs were fixed overnight at 4°C in Davidson's fixative (PH0975, Phygene, Fuzhou, China). Lenses were extracted after removal of the cornea and iris. Then, gradient dehydration with ethanol was conducted in a tissue dehydrator (Histore PEARL, Leica Microsystems, Germany), and the eyes were embedded in paraffin. The paraffin-embedded retinas were then sectioned into 5 μm slices using a paraffin microtome (BIOCUT, Leica Microsystems, Germany). Retinal sections were immersed in ultrapure water at room temperature and flattened at 55°C for 10 s before being mounted on adhesive microscope slides (188105, Citoglas, Jiangsu, China) and dried in a slide dryer for 6 h. Hematoxylin–eosin (HE) staining was performed only on retinal sections passing through the optic nerve head (ONH). HE staining was carried out using an automatic stainer (Autostainer XL, ST5010, Leica Microsystems, Germany). The slides were sealed with neutral balsam and coverslips after HE staining, and images were captured using a 3D slide scanner (Pannoramic, 3D, Belgium). ImageJ software (https://imagej.net/ij/index.html) was used to statistically analyse the thickness of the outer nuclear layer (ONL) at a consistent magnification. The retinal section was also stained with a primary anti-RPE65 antibody (MA5-32633, Invitrogen, 1:100) and anti-human nuclear antigen antibody (Ab191181, Abcam, 1; 100). Subsequently, the sample was stained with a secondary anti-rabbit IgG H&L (Alexa Fluor 555) (ab150078, Abcam, 1:500) and anti-mouse IgG H&L (Alexa Fluor 488) (ab150113, Abcam, 1:500). The retinal section was observed using confocal laser microscopy.

### Statistical analysis

The data are presented as the means and standard deviations of each condition. Group comparisons were conducted via one-way analysis of variance, followed by *post hoc* tests. *Post hoc* tests were performed using Tukey's Honestly Significant Difference (HSD) test to identify which specific groups differed from each other. The Dunnett test was used specifically to compare the experimental and control groups. GraphPad Prism 9.0 software (GraphPad Software, Inc., La Jolla, CA) was used for data analysis and mapping. The threshold for statistical significance was set at a probability (*P*) value <0.05.

## Results

### Preparation and characterization of the surface of the peptide-grafted PAI hydrogels

Peptides derived from the laminin β4 chain (PMQKMRGDVFSP) and vitronectin (PQVTRGDVFTMP) have been previously reported to support hPSC pluripotency and the differentiation of hPSCs into cardiomyocytes [37, 44, 59]. These two previously reported peptide sequences were selected and optimized with specific peptide modifications to create novel peptide sequences, and the efficacy of these novel peptides in supporting hPSC differentiation into RPE cells on PAI hydrogels grafted with these peptides was evaluated. The peptides used in this study included sequences derived from the laminin β4 chain (LB2CK, KLB2CK) and vitronectin (VN2CK, KVN2CK), as well as 1:1 mixtures LB2CK/VN2CK and KLB2CK/KVN2CK (Figure 1). KLB2CK includes an additional lysine (K) at the first residue of LB2CK. KVN2CK includes an additional lysine (K) at the first residue of VN2CK. We aimed to enhance the adhesion of hiPSCs to the PAI hydrogel surface grafted with KLB2CK and KVN2CK by adding lysine (K), because the cells have a negative surface potential, typically approximately −70 mV of resting potential, and hydrogels grafted with this modified peptide were compared with those grafted with LB2CK and VN2CK, respectively.

**Figure 2.**
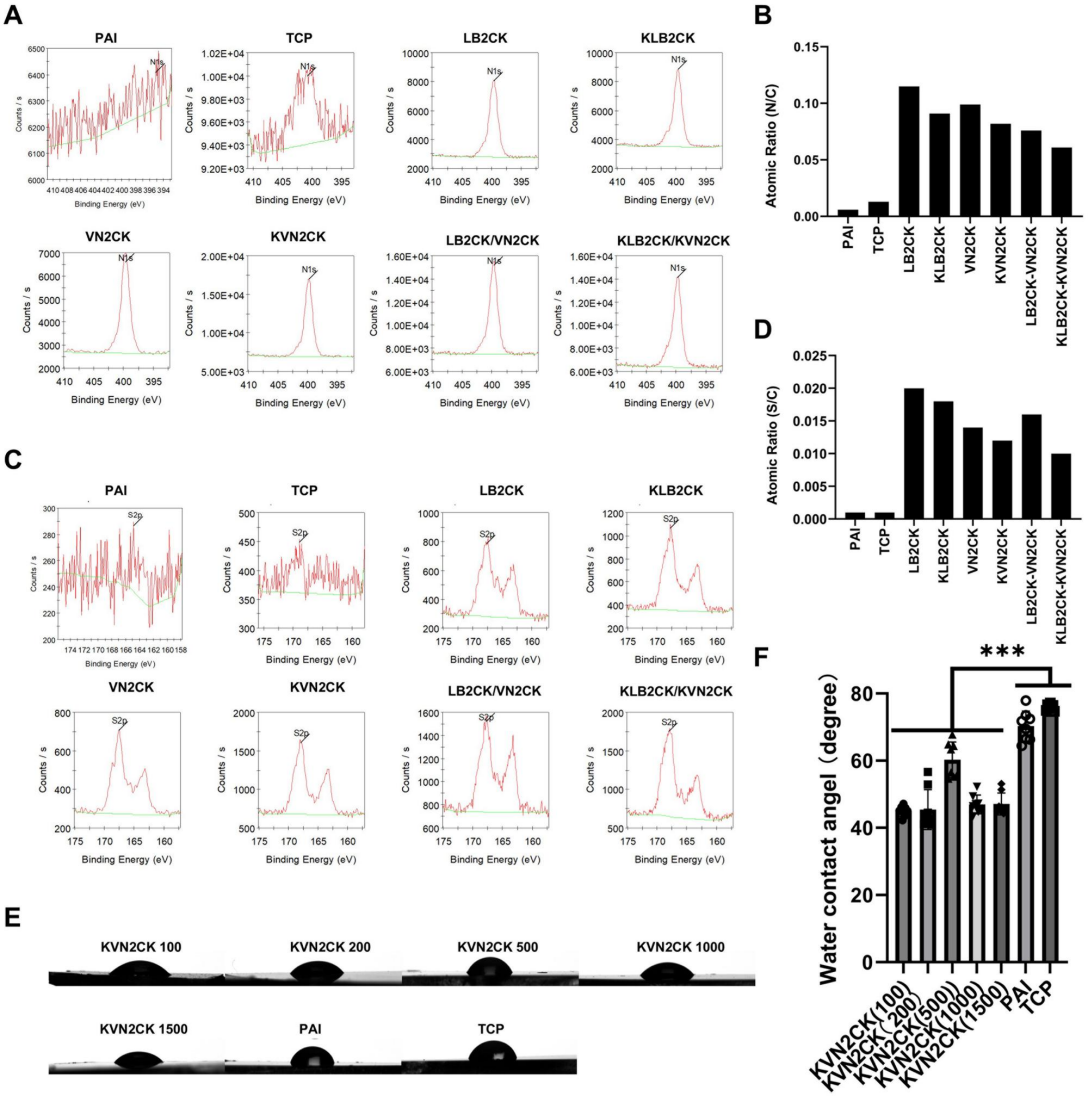
The physical properties of the peptide-grafted PAI hydrogel surface. (**A**) Nitrogen contents of distinct peptide-grafted PAI hydrogel surfaces prepared at a peptide concentration of 1000 μg/ml, as determined by XPS. (**B**) The nitrogen to carbon atomic ratio (N/C) of the peptide-grafted PAI hydrogel surface, the nonpeptide-grafted PAI hydrogel surface and the TCP surface. (**C**) Sulfur contents of distinct peptide-grafted PAI hydrogel surfaces prepared at a peptide concentration of 1000 μg/ml, as determined by XPS. (**D**) Sulfur to carbon atomic ratios (S/C) of the peptide-grafted hydrogel surface, the nonpeptide-grafted PAI hydrogel surface and the TCP surface. (**E**, **F**) Images of water droplets (**E**) and water contact angles (**F**) on the TCP surface, the nonpeptide-grafted PAI hydrogel surface and the peptide-grafted PAI hydrogel surface prepared with varying concentrations of KVN2CK.

X-ray photoelectron spectroscopy (XPS) analysis was conducted on the surface of the PAI hydrogels grafted with the designed peptides to ascertain whether the peptides were successfully grafted onto the PAI hydrogels. The atomic ratios of nitrogen to carbon (N/C) (Figure 2A and B) and of sulfur to carbon (S/C) (Figure 2C and D) on the surface of the PAI hydrogels grafted with each peptide were evaluated. The N/C ratio on the surface of the PAI hydrogels grafted with peptides was significantly greater than those of the PAI hydrogels without peptide grafting and TCP dishes. A similar tendency toward a high S/C ratio on the surface of the PAI hydrogels grafted with the peptide was observed compared with those of the PAI hydrogels without peptide grafting and TCP dishes. Because no nitrogen or sulfur atoms are present in the PAI hydrogels, the increased N1s content indicates successful grafting of peptides on the surface of the PAI hydrogels (Figure 2B), and the increased S2p content suggests successful grafting of cysteine, which is present in the peptides used in this study (Figure 2D).

The hydrophilicity of the surface of a material influences cell adhesion, proliferation and biocompatibility [60]. Therefore, we evaluated the water contact angle of PAI hydrogels grafted with KVN2CK, which were prepared with several peptide concentrations (100, 200, 500, 1000 and 1500 µg/ml), to evaluate their hydrophilicity. The results demonstrated that even at a low peptide concentration of 100 μg/ml, the hydrophilicity of the hydrogel surface was significantly increased, and there was no dependence of the water contact angle on the peptide concentration for PAI hydrogels grafted with KVN2CK at peptide concentrations ranging from 100 to 1500 µg/ml (Figure 2E and F).

### Optimal peptide and peptide concentration for the preparation of peptide-grafted hydrogels for hiPSC culture

**Figure 3.**
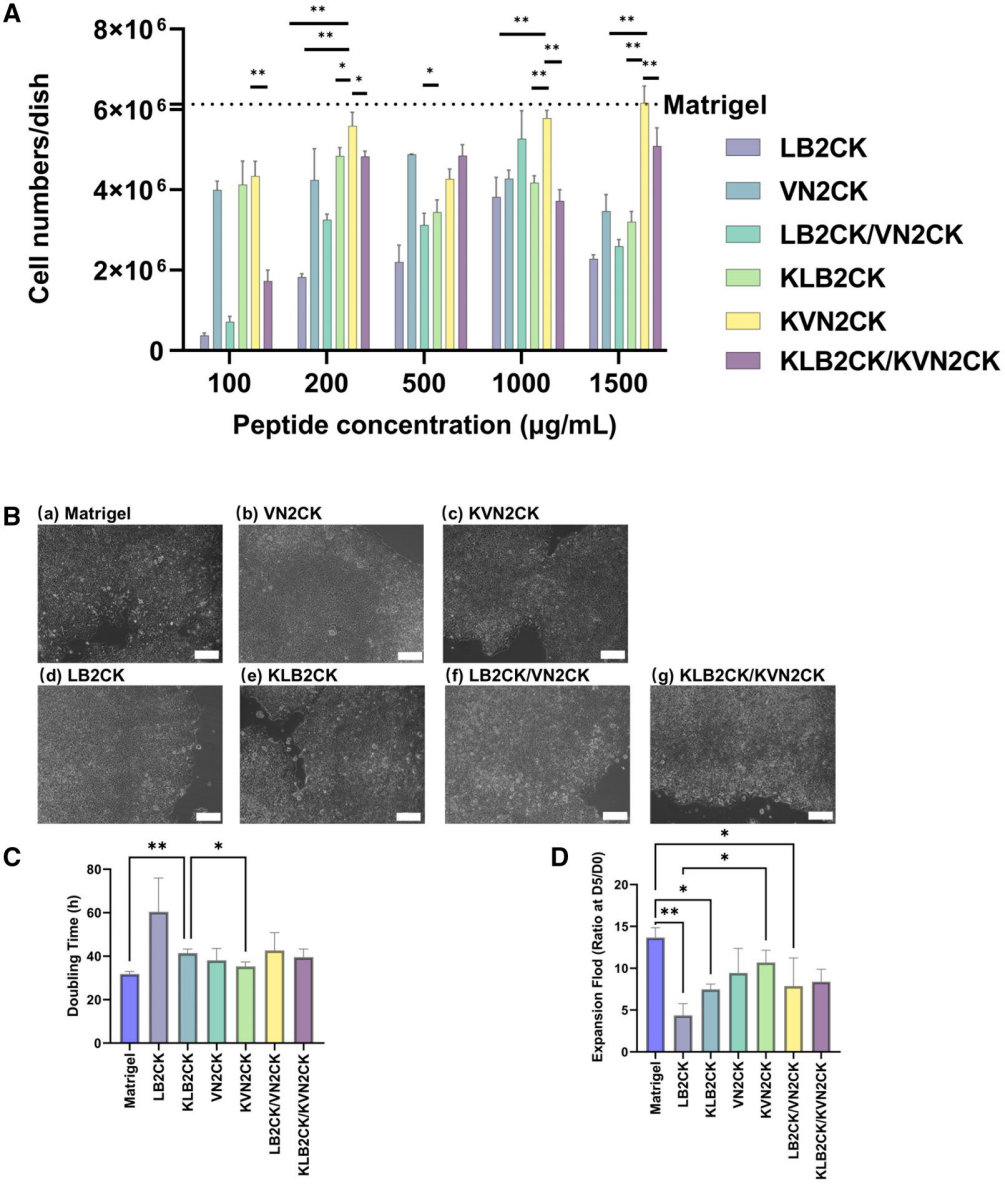
Attachment and survival of hiPSCs (HPS0077) on different peptide-grafted PAI hydrogel surfaces. (**A**) The number of cells harvested after 5 days of growth on peptide-grafted PAI hydrogel surfaces prepared with various peptide concentrations. (**B**) Microscopy images of hiPSCs grown for 5 days on Matrigel-coated surface (**a**) and peptide-grafted PAI hydrogel surfaces prepared with a peptide concentration of 1000 µg/ml (**b**–**g**). Scale bar: 100 µm. (**C**) Fold expansion of hiPSCs on Matrigel-coated surface and several peptide-grafted PAI hydrogel surfaces prepared with a peptide concentration of 1000 µg/ml. (**D**) Doubling time of hiPSCs cultured on Matrigel-coated surface and several peptide-grafted PAI hydrogel surfaces prepared with a peptide concentration of 1000 µg/ml. *P < 0.05; **P < 0.01.

The hiPSC cell line HPS0077 was cultured on PAI hydrogel surfaces grafted with four different peptides (VN2CK, KVN2CK, LB2CK and KLB2CK) and two peptide mixtures (LB2CK/VN2CK (1:1) and KLB2CK/KVN2CK (1:1)) at different peptide concentrations. These experiments were performed to assess the ability of peptide-grafted PAI hydrogel surfaces to support the long-term self-renewal of hiPSCs. Hydrogel surfaces generated with peptide concentrations of 100, 200, 500, 1000 and 1500 µg/ml were prepared to investigate the optimal peptide concentration for hiPSC culture. LB2CK- and LB2CK/VN2CK-grafted PAI hydrogels prepared with low peptide concentrations (e.g. 100 µg/ml) could not support hiPSC adhesion, as shown in Figure 3A and Supplementary Figure S1A. While the KLB2CK-grafted PAI hydrogel surface supported hiPSC adhesion, hiPSC colonies with rough edges were found on the hydrogel surface 5 days after cell inoculation (Supplementary Figure S1B). Notably, hiPSCs proliferated on VN2CK-, KLB2CK- and KVN2CK-grafted PAI hydrogels prepared with low peptide concentrations, such as 100 μg/ml.

The hiPSCs cultured on VN2CK-, KLB2CK- and KVN2CK-grafted PAI hydrogels prepared with 1000 μg/ml peptides exhibited a favorable colony morphology; these cells displayed the typical undifferentiated morphology required for colony formation, formed compact colonies with distinct edges and had a high nucleus–cytoplasm ratio, which was comparable to that of the Matrigel control group (Figure 3B). These findings suggest that the peptide-grafted PAI hydrogels prepared with 1000 µg/ml peptide represent the optimal cell culture biomaterials for supporting hiPSC adhesion and expansion under xeno-free culture conditions.

To quantify the growth efficiency of hiPSCs cultured on the peptide-grafted PAI hydrogel surface prepared with 1000 µg/ml peptide, the doubling time (Figure 3C) and fold expansion (Figure 3D) of hiPSCs on six different peptide-grafted hydrogel surfaces and a Matrigel-coated surface were calculated after 5 days of culture. The extent of cell expansion on hydrogel surfaces grafted with peptides derived from vitronectin (VN2CK: 9.4-fold, KVN2CK: 10.7-fold) was greater than that on surfaces grafted with peptides derived from the laminin β4 chain (LB2CK: 4.4-fold, KLB2CK: 7.5-fold). These data demonstrate that peptide-grafted hydrogel surfaces with lysine as the first amino acid in the sequence exhibited cell expansion efficiency superior to that of PAI hydrogel surfaces grafted with peptides in which lysine was not the first amino acid (Figure 3C and D). This result can be attributed to the presence of lysine as the first amino acid in the peptide sequence, which provides two amino groups that can flexibly link with the carboxyl groups of PAI hydrogels. Moreover, the optimal peptide type, KVN2CK, demonstrated expansion fold and doubling times comparable to those of the Matrigel surface (*P* < 0.05) (Figure 3C and D). The numbers of hiPSCs (HPS0077) present after culture on KVN2CK-grafted PAI hydrogels for five passages are also shown in Supplementary Figure S1C, and the results indicate that hiPSCs can be successfully cultivated on KVN2CK-grafted PAI hydrogels for a long period.

### Characterization of hiPSCs cultured on PAI hydrogels grafted with the optimal peptide and peptide concentration

**Figure 4.**
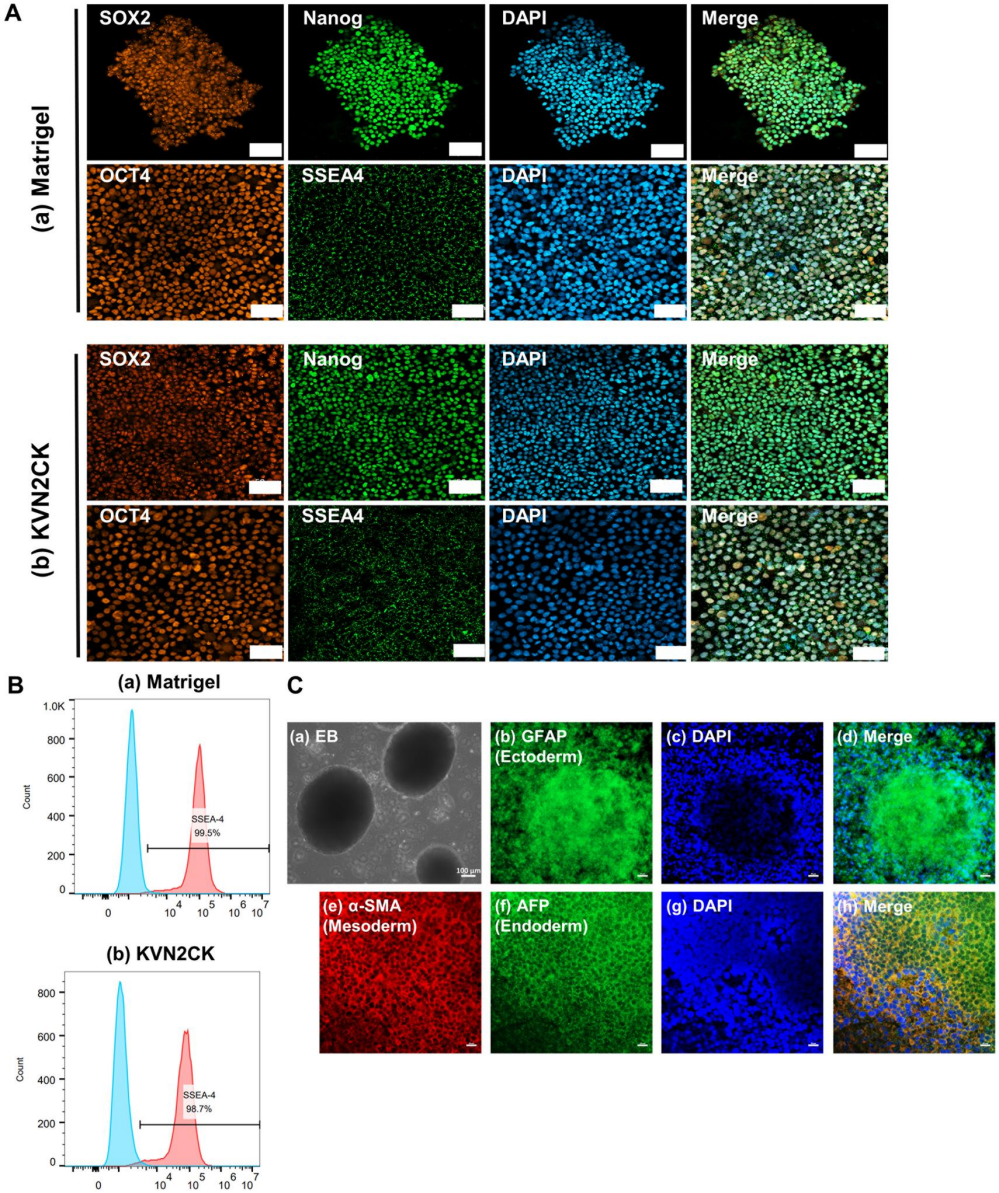
Evaluation of the pluripotency and differentiation capacity of hiPSCs (HPS0077) cultured on peptide-grafted PAI hydrogel surfaces. (**A**) Immunohistochemical staining for the pluripotency biomarkers SOX2 (red), NANOG (green), OCT4 (red) and SSEA-4 (green) in hiPSCs cultured on (**a**) Matrigel-coated surface and (**b**) KVN2CK-grafted PAI hydrogel surfaces prepared with a peptide concentration of 1000 µg/ml for five days. DAPI staining shows the cell nuclei (blue). Scale bar: 50 µm. (**B**) Flow cytometry histograms illustrating the expression of the pluripotency marker SSEA-4 in hiPSCs cultured on the (**a**) Matrigel-coated surface and (**b**) KVN2CK-grafted PAI hydrogel surface prepared with a peptide concentration of 1000 µg/ml for five days. (**C**) (**a**) Representative images of EBs differentiated from hiPSCs. Scale bar: 100 µm. (**b**–**h**) Immunofluorescence images of EBs derived from hiPSCs cultured on the KVN2CK-grafted PAI hydrogel surface prepared with a peptide concentration of 1000 µg/ml. Expression of markers of cells derived from the ectoderm (**b**, GFAP, green), mesoderm (**e**, α-SMA, red) and endoderm (**f**, AFP, green). DAPI (**c** and **g**, blue) was used for nuclear staining. Photos (**d**) and (**h**) were created by merging (**b**)–(**c**) and (**e**)–(**g**), respectively. Scale bar: 20 µm.

hiPSCs have the potential to differentiate into cells derived from three germ layers. The maintenance of pluripotency in hiPSCs is a pivotal criterion in the assessment of novel surfaces for hiPSC culture. The specific expression of protein markers can be used to identify the specific physiological functions of cells and can serve as a marker for identifying different cell types. The expression of the pluripotency markers OCT-4, SOX2, SSEA-4 and NANOG on the hiPSCs was evaluated via immunohistochemical staining after the hiPSCs were cultured for 5 days on each peptide-grafted PAI hydrogel (Figure 4A and Supplementary Figure S1D) [61]. The hiPSCs highly expressed pluripotency marker proteins, even after they were cultured on the peptide-grafted PAI hydrogels.

The expression of the pluripotency markers SSEA-4, OCT4, SOX2 and NANOG on hiPSCs was also evaluated via flow cytometry after the hiPSCs were cultured on KVN2CK-grafted PAI hydrogels prepared with 1000 µg/ml KVN2CK or Matrigel-coated dishes (control experiments) for 5 days and the results are shown in Figure 4B and Supplementary Figure S2. A total of 98.7% and 99.5% of the hiPSCs expressed SSEA4 after culture on KVN2CK-grafted PAI hydrogels and Matrigel-coated dishes, respectively (Figure 4B). Furthermore, over 95% and 91% of the hiPSCs expressed OCT4, SOX2 or NANOG after culture on KVN2CK-grafted PAI hydrogels and Matrigel-coated dishes, respectively (Supplementary Figure S2). These data indicated that hiPSCs maintained high expression of pluripotency markers even after culture on KVN2CK-grafted PAI hydrogels.

Importantly, hiPSCs have the ability to differentiate into cells derived from three germ layers and can develop into any type of cell in the body. The embryoid (EB) formation method was used to evaluate the differentiation ability of hiPSCs (HPS0077) cultured on the KVN2CK-hydrogel surface *in vitro*, and the results are shown in Figure 4C. Markers of ectoderm (GFAP; glial fibrillary acidic protein), mesoderm (α-SMA; α-smooth muscle actinin) and endoderm (AFP; α-fetoprotein) differentiation were detected via an immunofluorescence assay (Figure 4C).

### The peptide-grafted PAI hydrogel surface supports the differentiation of hiPSCs into RPE cells

**Figure 5.**
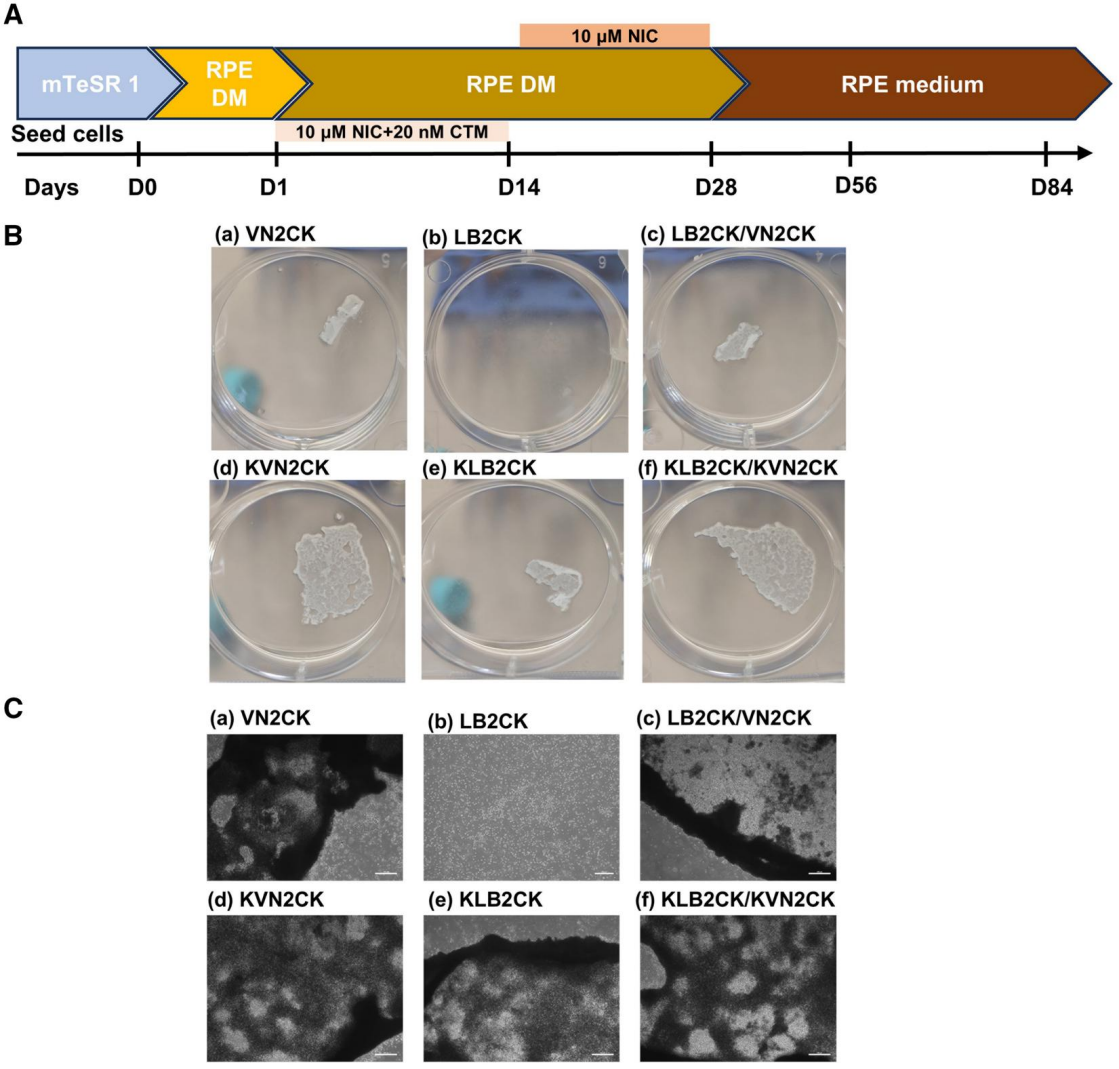
hiPSCs (HPS0077) differentiated into RPE cells via the NIC84 differentiation protocol. (**A**) Timeline of the NIC84 differentiation protocol. (**B**) Images of hiPSCs differentiated on the surfaces of several peptide-grafted PAI hydrogels prepared at a peptide concentration of 1000 µg/ml after 28 days of induction into RPE cells. (**C**) Micrographs of hiPSCs differentiated on the surfaces of several peptide-grafted PAI hydrogels prepared at a peptide concentration of 1000 µg/ml after 28 days of induction into RPE cells. Scale bars: 200 µm.

The goal of this study was to achieve large-scale production of GMP-grade hiPSC-derived RPE cells on synthetic peptide-grafted PAI hydrogels under xeno-free cell culture conditions. We selected the NIC differentiation protocol [45, 46, 62] for the differentiation of hiPSCs (HPS0077 and MIX2) into RPE cells (Figure 5A); this protocol is primarily based on the modified protocol developed by Maruotti *et al.* [45]. The NIC differentiation protocol includes the transition of pluripotent hiPSCs cultured in mTeSR1 medium into DM on the first day of differentiation. In the initial attempt to induce hiPSC differentiation into RPE cells, 50 nM CTM was used from the second day to the 14th day of differentiation, in accordance with the original differentiation protocol [45]. However, these conditions generated a notable increase in cell death in this study. This can be attributed to the difference in the tolerance of each hPSC line to different CTM concentrations [45]. Therefore, we reduced the concentration of CTM from 50 to 20 nM in the NIC protocol in this study.

The majority of cells differentiated from hiPSCs (HPS0077) cultured on the LB2CK-grafted PAI hydrogel surface were detached, and minimal cell adhesion was observed on VN2CK- and LB2CK/VN2CK-grafted PAI hydrogel surfaces by the 28th day of differentiation. Furthermore, the surface area of the cells retained on the surface of peptide-grafted PAI hydrogels in which the peptide had KGG at the N-terminus was greater than that on the surface of peptide-grafted PAI hydrogels in which the peptide did not have KGG at the N-terminus (Figure 5B). This phenomenon is also proven by the micrographs taken in each peptide-grafted PAI hydrogel (Figure 5C). Hydrogels supporting relatively robust adhesion (e.g. KVN2CK, KLB2CK/KVN2CK) maintained partial of the confluent RPE monolayers in differentiation with less rolling up of cell edges. While hydrogels with suboptimal adhesion properties (e.g. LB2CK) exhibited near-complete cell detachment. We speculated that peptides with positive lysine (K) residues inserted at the N-terminus exhibit enhanced cell adhesion capabilities, which contributes to long-term cell adhesion during differentiation into RPE cells.

Microscopic observation of differentiated cells on the Matrigel and KVN2CK-grafted PAI hydrogel surfaces on the 42nd day of differentiation revealed the presence of a significantly increased number of pigmented cells (Supplementary Figure S3A), accompanied by an increase in pigment cell area as the differentiation time increased. The production of pigment cells via hiPSC (HPS0077) differentiation was exclusively observed on KVN2CK-grafted PAI hydrogel surfaces and Matrigel-coated dish surfaces in the later stages of differentiation (Supplementary Figure S3B). In contrast, the cells that had undergone differentiation into RPE cells on the KLB2CK- and KLB2CK/KVN2CK-grafted PAI hydrogels exhibited complete detachment from the surface (Supplementary Figure S3B).

**Figure 6.**
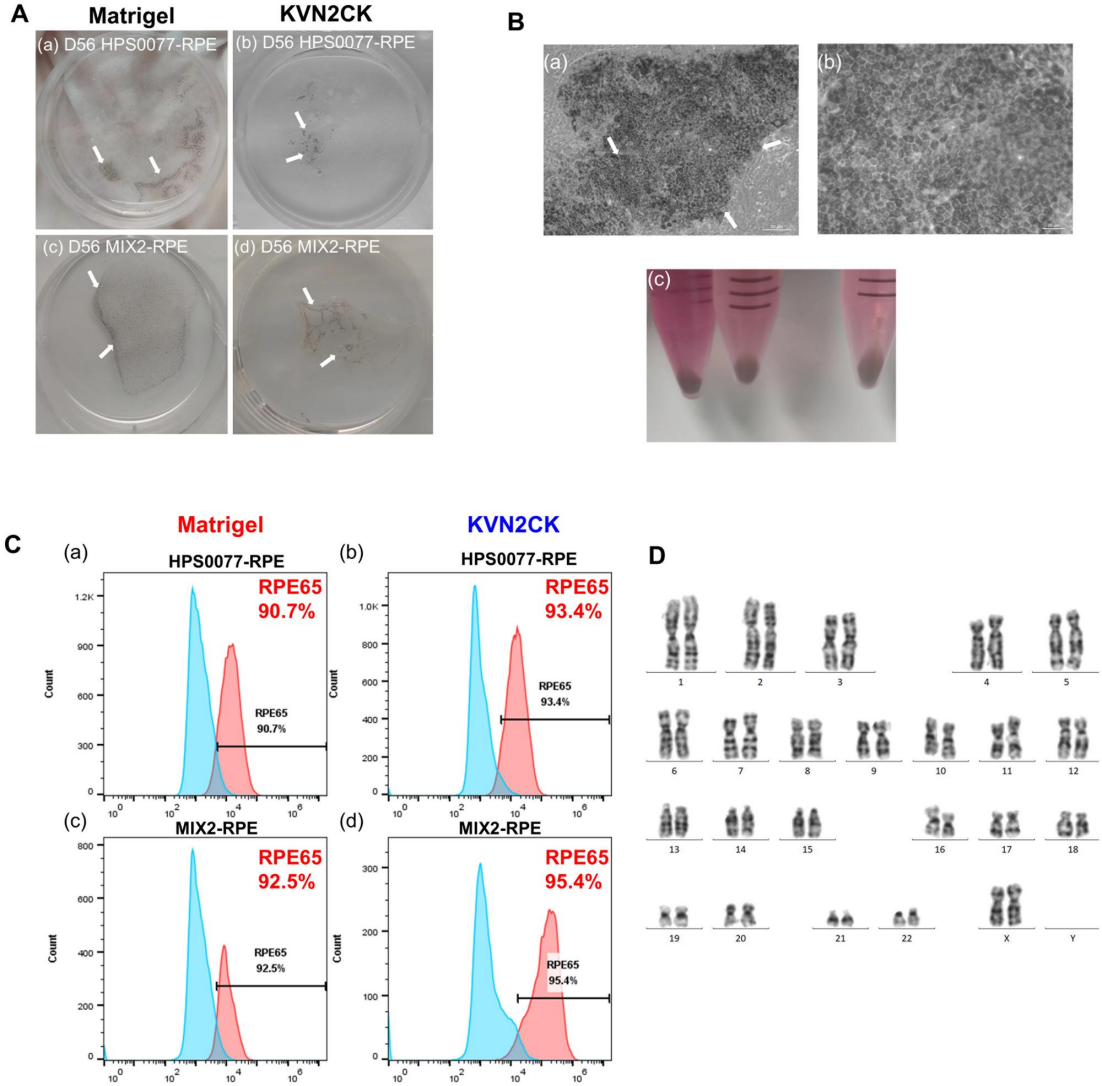
Differentiation of two hiPSC lines (HPS0077 and MIX2) into RPE cells via the NIC84 protocol on the KVN2CK-grafted PAI hydrogel surface and a Matrigel-coated surface. (**A**) Photos of pigment cells among RPE cells differentiated from HPS0077 (**a**, **b**) and MIX2 (**c**, **d**) hiPSCs cultured on a Matrigel-coated surface (**a**, **c**) and a KVN2CK-grafted PAI hydrogel surface (**b**, **d**) prepared at a peptide concentration of 1000 µg/ml after 56 days of induction. (**B**) Microscopy images of hiPSC-derived RPE cells. (**a**) Morphology of polygonal pigmented cells and (**b**) magnified image cultured on a KVN2CK-grafted PAI hydrogel surface prepared at a peptide concentration of 1000 µg/ml after 84 days of induction. Scale bars are 50 µm (**a**) and 20 µm (**b**). (**c**) Photo of collected mature pigmented cells from hiPSC-derived RPE cells. (**C**) Flow cytometry analysis of the expression of the RPE-specific protein RPE65 on RPE cells derived from HPS0077 (**a**, **b**) and MIX2 (**c**, **d**) hiPSCs cultured on Matrigel-coated surfaces (**a**, **c**) and KVN2CK-grafted PAI hydrogel surfaces (**b**, **d**) prepared at a peptide concentration of 1000 µg/ml. (**D**) Karyotype analysis of RPE cells derived from HPS0077 hiPSCs after 84 days of differentiation on the KVN2CK-grafted hydrogel surface prepared at a peptide concentration of 1000 µg/ml.

We also conducted RPE cell differentiation using another hiPSC cell line, MIX2, to demonstrate the reproducibility of hiPSC differentiation into RPE cells on KVN2CK-grafted PAI hydrogel surfaces. Notably, more pigment spots (indicating RPE cells) were generated from the MIX2 cells than from the HPS0077 cells after 42 days (Supplementary Figure S3A), although the degree of pigmentation in the cells differentiated from the MIX2 cells was comparable to that in the cells differentiated from the HPS0077 cells after 56 days (Figure 6A). We speculate that MIX2 cells, which are derived from amniotic fluid stem cells (fetal stem cells), exhibit higher cell viability and are more amenable to differentiation into RPE cells. This is probably because MIX2 cells are relatively young hiPSCs (with a passage number of approximately 30–40), whereas HPS0077 cells are older (with a passage number of more than 100). A substantial number of hexagonal retinal pigment epithelial cells were observed on KVN2CK-grafted PAI hydrogels by the 84th day of MIX2 differentiation following the manual removal of nonpigmented cells for the purposes of passaging and expansion (Figure 6B).

**Figure 7.**
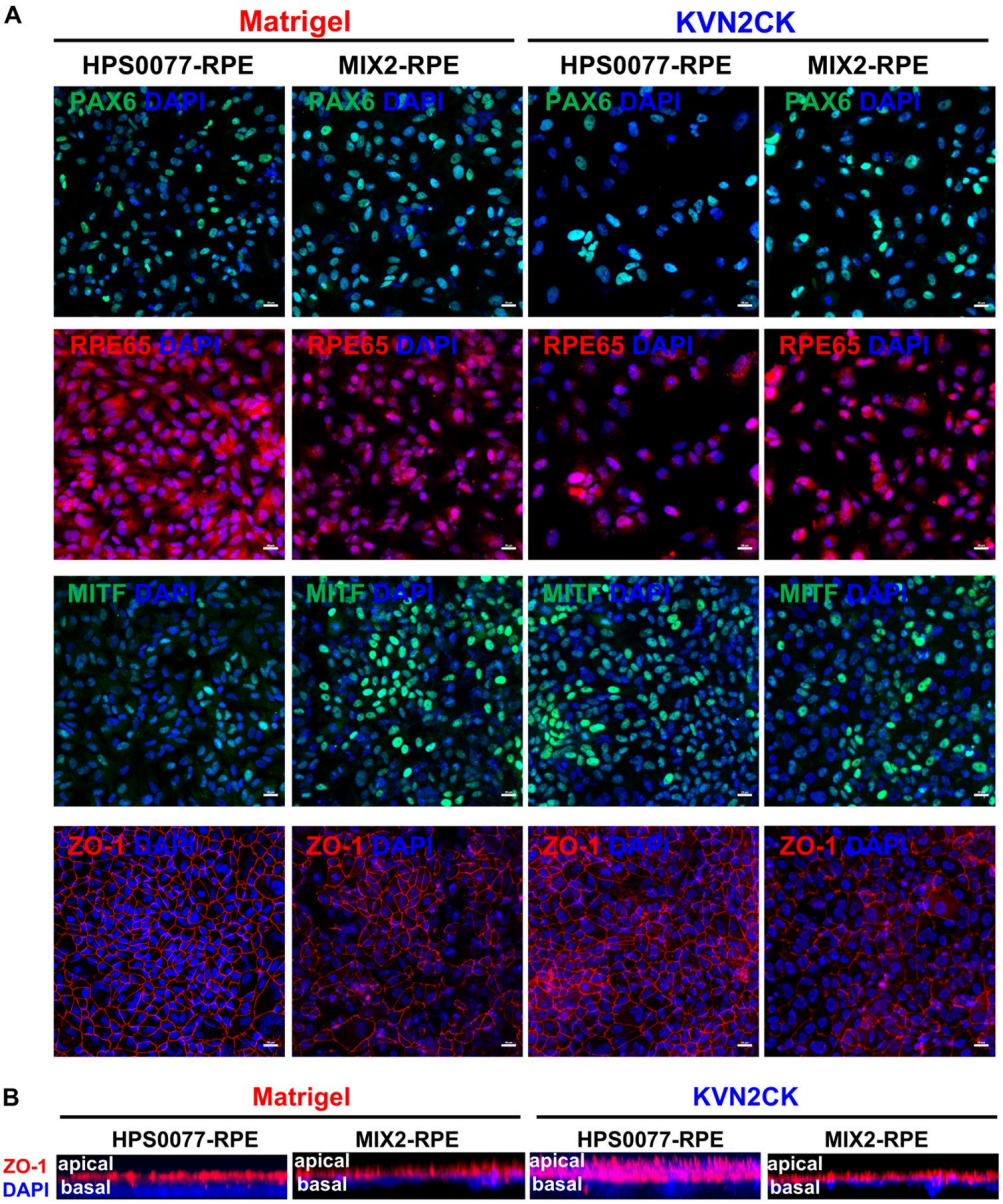
Immunohistochemistry images of RPE cells derived from HPS0077 and MIX2 hiPSCs on day 84 of differentiation, analysed by confocal laser microscopy. (**A**) Expression of PAX6 (green), ZO-1 (red), MITF (green) and RPE65 (red) on hiPSCs (HPS0077 and MIX2)-derived RPE cells, when the cells were cultured on a Matrigel-coated surface and the KVN2CK-grafted PAI hydrogel surface prepared at a peptide concentration of 1000 µg/ml. Scale bar: 20 µm. (**B**) The cross section of immunohistochemistry images of hiPSCs (HPS0077 and MIX2)-derived RPE cells, which were evaluated on the same sample and site analysed in the picture of (**a**). The same scale to the picture of (**a**).

Immunohistochemical staining was performed for HPS0077-derived RPE cells and MIX2-derived RPE cells cultured on KVN2CK-grafted PAI hydrogel surfaces and Matrigel-coated dishes, and the following markers were used to evaluate RPE cells: PAX6, ZO-1, RPE65 and MITF (Figure 7). PAX6, ZO-1, RPE65 and MITF were highly expressed on RPE cells derived from both HPS0077 and MIX2 hiPSCs (Figure 7A) after culture on KVN2CK-grafted PAI hydrogels and Matrigel-coated dishes. The cross section of hiPSC-derived RPE cells evaluated from confocal laser microscopy is shown in Figure 7B, which was taken from the site measured in Figure 7A. The apical and basal sides of the hiPSC-derived RPE monolayers are clearly found in Figure 7B.

The purity of hiPSC-derived RPE cells was assessed on the basis of RPE65 expression via flow cytometry, as RPE65 is associated with visual cycle function. The cells derived from both hiPSC cell lines (HPS0077 and MIX2) presented high expression of RPE65, with expression in more than 90% of cells cultured on KVN2CK-grafted PAI hydrogels and Matrigel-coated dishes. Especially, the RPE cells derived from MIX2 cells cultured on KVN2CK-grafted PAI hydrogel surfaces presented high RPE65 expression (95.4%) (Figure 6C). These findings support the hypothesis that KVN2CK-grafted PAI hydrogels are excellent xeno-free cell culture biomaterials for successful differentiation of hiPSCs into mature RPE cells, thereby providing a cellular source of hiPSC-derived RPE cells for subsequent subretinal transplantation into RCS rats in the following experiments. Furthermore, hiPSC-derived RPE cells harvested from the surface of KVN2CK-grafted PAI hydrogels maintained their karyotype integrity after ten passages on the KVN2CK-grafted hydrogel surface (Figure 6D), and this pattern was observed for both HPS0077-derived RPE cells (Figure 6D) and MIX2-derived RPE cells (Supplementary Figure S4), indicating that the KVN2CK-grafted PAI hydrogel surface supported long-term *in vitro* culture of hiPSC-derived RPE cells without genetic modification.

The secretion of several growth factors (VEGF and PEDF) by HPS0077-derived RPE cells cultured on KVN2CK-grafted PAI hydrogel surface and Matrigel-coated dishes was also evaluated. The cultivation medium of each cell condition was obtained from confluent cell samples at two days after the cultivation medium was changed to estimate the secretion of those growth factors using enzyme-linked immunosorbent assay (ELISA). All hiPSC-derived RPE cells secreted human VEGF and PEDF, but they secret almost same degrees among hiPSCs (HPS0077) cultured on KVN2CK-grafted PAI hydrogel surface and Matrigel-coated dishes (Supplementary Figure S5). The secreted amount of these growth factors is close to the amount reported by our previous work [57]. These results indicate that any hiPSC-derived RPE cells cultured on KVN2CK-grafted PAI hydrogel surface and Matrigel-coated dishes show healthy function of hiPSC-derived RPE cells in this study.

### Validation of the functionality of hiPSC-derived RPE cells cultured on KVN2CK-grafted hydrogels *in vivo*

hiPSC-derived RPE cells generated from HPS0077 cells and MIX2 cells via differentiation on a KVN2CK-grafted PAI hydrogel surface or a Matrigel-coated dish surface were transplanted into the subretinal space of retinal degeneration model rats (RCS rats). Visual function improvement on RCS rats was evaluated to assess the efficacy of hiPSC-derived RPE cells differentiated on peptide-grafted hydrogel surfaces in comparison to previously reported hiPSC-derived RPE cells differentiated on Matrigel-coated dish surfaces [63, 64].

RCS rats are commonly used as a retinal degeneration animal model and they have been used for RPE cell transplantation [65, 66] because of a mutation in the *Mertk* gene, which impairs the phagocytic function of RPE cells [67]. hiPSC-derived RPE cells were labeled with CellTracker™ CM-DiI dye and suspended in 2 µl of PBS at a concentration of 5 × 10^4^ cells/μl for transplantation. The apoptosis of photoreceptor cells in RCS rats commences during the third week postnatally, with the vast majority of photoreceptor cells undergoing apoptosis by the age of 2–3 months [68–70]. Therefore, four distinct types of hiPSC-derived RPE cells (RPE cells derived from HPS0077 cells and MIX2 cells, which were each differentiated on KVN2CK-grafted PAI hydrogels and Matrigel-coated dishes) were transplanted into the subretinal space of the right eye in RCS rats (five RCS rats per each condition) during the early stage of photoreceptor cell apoptosis (21 days after birth), to generate the experimental group. The control group consisted of RCS rats that received an equal volume of PBS injected into the subretinal space of the right eye.

**Figure 8.**
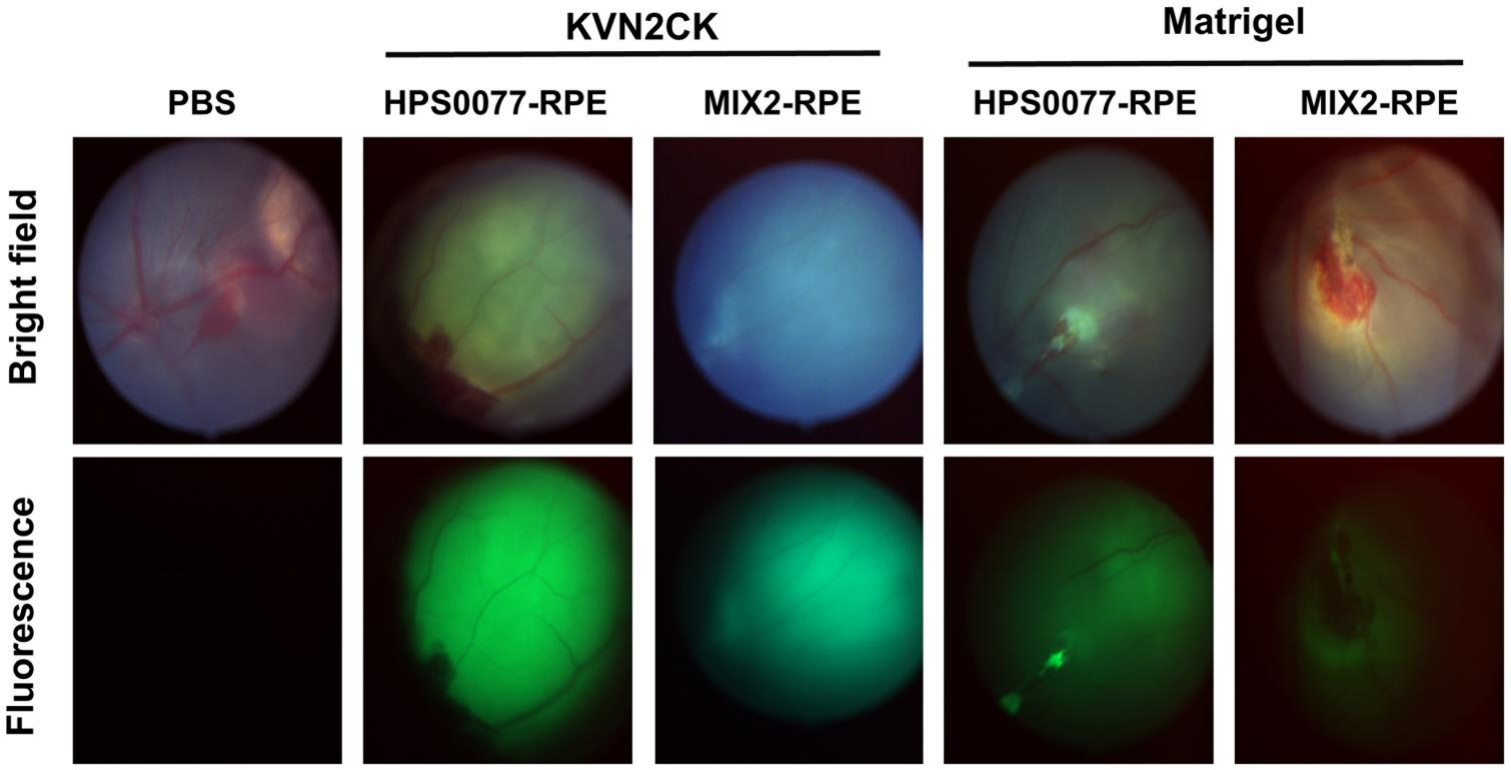
Evaluation of visual function in Royal College of Surgeons (RCS) rats following cell therapy. Fundus photographs of RCS rats following subretinal injection of hiPSC-derived RPE cells cultured on KVN2CK-grafted PAI hydrogel surfaces prepared at a peptide concentration of 1000 µg/ml and Matrigel-coated surfaces; images were captured via bright field and fluorescence microscopy. The successful transplantation of cells is indicated by the presence of strong fluorescence at the transparent retinal site. In contrast, the control group that received PBS exhibited no fluorescence.

Fundus photography was performed to observe the fundus of the transplantation site in the RCS rats and to assess the quality of the transplanted hiPSC-derived RPE cells during the surgical procedure. The group in which the hiPSC-derived RPE cells were stained with CellTracker™ CM-DiI dye and then transplanted exhibited a notable elevation of the retina and green fluorescence at the elevated subretinal site on fundus photography (Figure 8). In contrast, the sham surgery group injected with only PBS exhibited a significant elevation of the retina, but no green fluorescence was detected. Furthermore, no evidence of tumor formation or abnormal cell proliferation was observed during this study.

The optomotor response (OMR) score is defined as the ratio of consistency and inconsistency between the body/head movements of RCS rats and the movement on the screen surrounding the system. A threshold of 1.0 has been established for sensory perception. Animals with OMR scores below 1.0 are deemed incapable of perceiving stimuli at the investigated spatial frequency [58]. The OMR scores of RCS rats in the fourth week after subretinal transplantation of hiPSC-derived RPE cells differentiated on KVN2CK-grafted PAI hydrogels and Matrigel-coated dishes are shown in Supplementary Figure S6. At 4 weeks after subretinal transplantation, the RCS rats were unable to stand in a normal position on the central platform of the box because of the small area of the box. Consequently, the OMR scores for the late post-injection period (more than 4 weeks after subretinal transplantation) could not be obtained in this study. The OMR scores of RCS rats subjected to cell transplantation were collected and recorded 4 weeks after injection (Supplementary Figure S6A). The rats that had received transplanted cells presented higher OMR scores (>1.0) at 0.1 and 0.2 c/g frequencies than the rats in the sham surgery group did (Supplementary Figure S6B and C). Consistent with these findings, several studies have reported that significant OMR responses in rats were observed within the 0.1–0.2 c/g range following subretinal transplantation of hPSC-derived RPE cells [58, 71]. However, no significantly statistical differences between the cell transplantation group and the sham group were identified in this study (*P* > 0.05).

**Figure 9.**
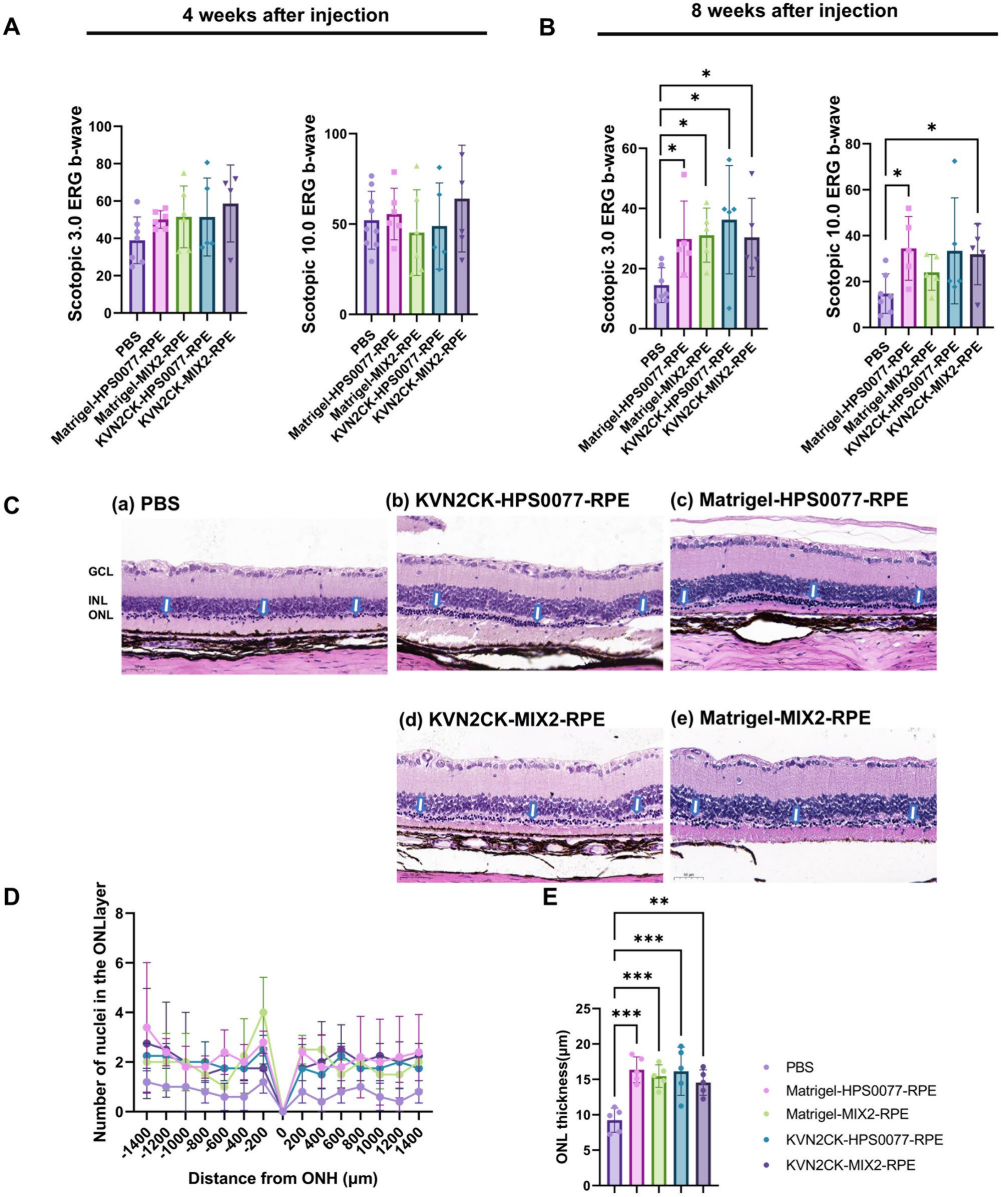
Evaluation of the effects of subretinal transplantation of hiPSC-derived RPE cells on RCS rats. (**A**, **B**) The *b*-wave amplitude of RCS rats at 3.0 cd s/m^2^ (right figure) and 10.0 cd s/m^2^ (left figure) under dark conditions 4 weeks (**A**) and 8 weeks (**B**) after subretinal transplantation of RPE cells derived from HPS0077 and MIX2, which were cultured on a KVN2CK-grafted PAI hydrogel surface prepared at a peptide concentration of 1000 µg/ml and a Matrigel-coated surface. (**C**) Representative hematoxylin–eosin (HE)-stained images of cross sections of retinas from RCS rats 8 weeks after the transplantation of PBS (**a**) and HPS0077-derived RPE cells (**b**, **c**), which were cultured on KVN2CK-grafted PAI hydrogel surfaces prepared at a peptide concentration of 1000 µg/ml (**b**) and Matrigel-coated surfaces (**c**), and MIX2-derived RPE cells (**d**, **e**), which were cultured on KVN2CK-grafted PAI hydrogel surfaces prepared at a peptide concentration of 1000 µg/ml (**d**) and Matrigel-coated surfaces. GCL: ganglion cell layer; INL: inner nuclear layer; ONL: outer nuclear layer. The arrows indicate ONL sites. (**D**) The number of layers of cell nuclei in the ONL at 14 points in RCS rats transplanted with PBS, HPS0077-derived RPE cells cultured on the KVN2CK-grafted PAI hydrogel surface prepared at a peptide concentration of 1000 µg/ml (KVN2CK-HPS0077-RPE), MIX2-derived RPE cells cultured on the KVN2CK-grafted PAI hydrogel surface prepared at a peptide concentration of 1000 µg/ml (KVN2CK-MIX2-RPE), HPS0077-derived RPE cells cultured on the Matrigel-coated surface (Matrigel-HPS0077-RPE), and MIX2-derived RPE cells cultured on the Matrigel-coated surface (Matrigel-MIX2-RPE) at 8 weeks post-transplantation. (**E**) The ONL thickness of RCS rats transplanted with PBS, KVN2CK-HPS0077-RPE, KVN2CK-MIX2-RPE, Matrigel-HPS0077-RPE or Matrigel-MIX2-RPE cells at 8 weeks post-injection. Statistical significance levels are indicated as *P < 0.05, **P < 0.01 or ***P < 0.01.

ERG was used to evaluate the visual function of RCS rats to further investigate the effects of subretinal transplantation of hiPSC-derived RPE cells after culture on KVN2CK-grafted PAI hydrogels and Matrigel-coated dishes (Figure 9A and B). The ERG results demonstrated that compared with the noninjected control rats, the RCS rats subjected to the transplantation of hiPSC-derived RPE cells cultured on both KVN2CK-grafted PAI hydrogels and Matrigel-coated dishes presented ERG responses under dark field stimulation intensities of 3.0 and 10.0 cd s/m^2^ at 4 weeks post-injection (Figure 9A). Nevertheless, the difference in the *b*-wave amplitude between the cell transplantation group and the sham surgery group injected with PBS was not statistically significant (*P* > 0.05) at 4 weeks post-injection. The ERG responses of RCS rats that received hiPSC-derived RPE cells from four different sources exhibited similar patterns (*P* < 0.05). The *b*-wave amplitude was markedly diminished in the sham surgery group at 8 weeks post-injection (Figure 9B), whereas a strong ERG response was observed in RCS rats transplanted with RPE cells derived from HPS0077 and MIX-2 hiPSCs cultured on KVN2CK-grafted PAI hydrogels and Matrigel-coated dishes, suggesting that RPE cell transplantation could restore the visual function of RCS rats even during the late stage (8 weeks post-injection) (*P* < 0.05).

Our findings indicated that PBS injection exhibited some degree of efficacy in restoring visual function in RCS rats during the initial phase (4 weeks post-injection) but not in the late stage (8 weeks post-injection) in this study. This effect may be attributed to the ability of subretinal injection to stimulate the release of growth factors that regulate retinal neovascularization and inflammation in RPE or other cells [72, 73].

The cell bodies of photoreceptor cells are located within the ONL of the retina. An increase in the thickness of the ONL is indicative of a greater number of photoreceptor cells. The eyeballs of RCS rats were removed for histological evaluation of the transplantation of hiPSC-derived RPE cells from different sources at 8 weeks post-injection (Figure 9C). The retinal thickness of ONL and the number of nuclei per column in the ONL were calculated in this study. A total of 14 positions, with each point at a 200 μm interval from the ONH, were selected for measurement for each section. The retinal thickness of ONL at each point and the average retinal thickness of ONL at all 14 points were evaluated and analysed as following the method established in our previous study [57]. The thickness of the ONL was significantly greater in the cell therapy group than in the PBS sham surgery group. A reduction in the thickness of the ONL and a decrease in the number of nuclear layers within the ONL were observed in the eyes of the rats in the sham surgery group, whereas the retinas of the rats subjected to hiPSC-derived RPE cell transplantation demonstrated maintenance of the ONL thickness (Figure 9D). Our observed ONL thickness in the transplanted eye indicates photoreceptor preservation but may slightly overestimate due to the limitations of our study, where we do not report complete evidence of transplanted RPE integration with host cells. The retinas of the rats subjected to transplantation of hiPSC-derived RPE cells after culture on KVN2CK-grafted PAI hydrogels and Matrigel-coated dishes presented thicker ONL than the sham surgery group did (Figure 9E) (*P* < 0.05), confirming that hiPSC-derived RPE cells differentiated on KVN2CK-grafted PAI hydrogel surfaces under xeno-free culture conditions and exerted a beneficial effect on the maintenance of retinal structure following transplantation.

The retinal sections transplanted with hiPSC-derived RPE cells, which were cultured on KVN2CK-grafted PAI hydrogel surface, were also stained with antibodies of human nuclei and RPE65 (Supplementary Figure S7). We observed a faint immune staining of human nuclei and human RPE cells even after 8 weeks of subretinal transplantation of hiPSC-derived RPE cells. These results indicate that some hiPSC-derived RPE cells, which were transplanted subretinally could survive even after post-injection of 8 weeks. These results indicate that visual functional improvement of RCS rats after transplantation of hiPSC-derived RPE cells could be based on the transplanted hiPSC-derived RPE cells in this study.

## Discussion

To date, a range of nonxenogeneic hPSC culture substrates, including human recombinant ECM proteins and their fragments, have been employed in clinical applications [34, 74]. However, further optimization of hPSC culture substrates is necessary for regenerative medical therapies based on stem cell therapies. This optimization process should include the establishment of stable culture conditions, good sterilization performance, reduced variation across experimental batches and reduced production costs to obtain high-quality and uncontaminated hPSC-derived cells. Therefore, we designed an artificially synthesized and well-defined stem cell culture substrate, peptide-grafted PAI hydrogels, which can facilitate hiPSC attachment, self-renewal and differentiation into specific cell lineages and serve as an alternative to the use of recombinant ECM protein-coated dishes. One goal of this study is to facilitate the future large-scale automation of stem cell production for clinical usage.

The coupled peptide sequences used here were screened and optimized in the initial stages of the study. The peptide-grafted PAI hydrogel surface with lysine as the first amino acid in the sequence achieved superior cell proliferation efficiency and hiPSC differentiation into RPE cells in comparison to the peptide-grafted PAI hydrogel surface in which lysine was not present in the first residue of the peptide. This observation can be attributed to the enhanced flexibility of the NH_2_ side chain of lysine, which may facilitate effective and flexible movement of the peptide to enable binding to the integrin receptor on hiPSCs.

The different hydrogel surfaces prepared in this study were evaluated for their ability to maintain the pluripotency of hiPSCs. Additional physical and chemical characteristics of the cell culture surface, including the elastic modulus, surface roughness, hydrophilicity and surface functional groups, also influence stem cell adhesion and proliferation [75]. These factors may contribute to further optimization of the peptide-grafted PAI hydrogels.

Our results indicate that the KVN2CK-grafted PAI hydrogel surface is conducive to the differentiation of hiPSCs into RPE cells. The production of RPE cells requires a long period, such as 84 days, and a cell culture substrate with high adhesive ability is thus required. The hiPSC-derived RPE cells were obtained exclusively on the KVN2CK-grafted PAI hydrogel surface, which presented the highest adhesive proliferation capacity in this study, indicating the potential of this cell culture material. Compared with the commercially available hiPSC cell line HPS0077, MIX2 cells exhibited enhanced suitability for RPE differentiation. This observation may be attributed to the fact that MIX2 cells were reprogrammed from fetal stem cells in amniotic fluid, which are more primitive than adult stem cells and exhibit a pluripotency profile more closely aligned with that of embryonic stem cells [56].

Our findings indicate that PBS also partially restored visual function in RCS rats at 4 weeks post-injection. Similarly, there have been reports that saline injections preserve photoreceptor viability for at least 2 months [76], and it has been demonstrated that temporary retinal detachment may induce photoreceptor rescue in RCS rats [76]. Similarly, in RDS mice, another animal model of retinal degeneration disease, subretinal injection of PBS resulted in decreased photoreceptor death and a reduction in the expression of the photoreceptor protein peripherin 2, which is mutated in RDS mice [77]. These phenomena may be explained by the activation of complement-related pathways.

Our study primarily focused on the development of peptide-grafted hydrogels as an alternative to Matrigel-coated dishes for the cell culture materials of differentiation of hPSCs into RPE cells [78, 79]. Typically, Matrigel-coated dishes have been used for hPSC culture and differentiation. Matrigel is the solubilized basement membrane matrix secreted by Engelbreth–Holm–Swarm (EHS) mouse sarcoma cells, which contains laminin, nidogen, collagen and heparan sulfate proteoglycans as well as growth factors such as TGF-beta and EGF [78, 79]. However, the exact composition of Matrigel varies from lot to lot. Especially, Matrigel is xeno-derived materials and not chemical defined components, which obstracts the usage of cell culture coating materials for clinical application. On the other hand, peptide-grafted PAI hydrogels developed in this study are xeno-free and chemical defined cell culture materials, which are preferable to use hPSC culture and differentiation for clinical application. These hydrogels provide a defined and tunable microenvironment that supports RPE maturation. However, for transplantation purposes, several additional factors must be considered, including mechanical stability, integration with host tissue, FDA approval and functional performance *in vivo*. As a limitation of the current study, we need to proceed evaluation of our hiPSC-derived RPE cells cultured on peptide-grafted PAI hydrogels using transepithelial resistance (TER), phagocytosis activity and melanin pigment determination using transmission electron microscopy (TEM) as long as long-term transplant analysis of hiPSC-derived RPE cells into RCS rats (beyond 8 weeks) in our future work.

In the literature [80, 81], different RPE transplantation approaches have been explored, including cell suspensions, cell monolayers on scaffolds and bioengineered sheets. While peptide-grafted hydrogels may support monolayer formation and enhance RPE differentiation, further modifications would be necessary to ensure that the hydrogel meets the mechanical and biochemical requirements for transplantation. For example, crosslinking strategies or hybrid scaffold designs could improve the structural integrity and handling properties of the hydrogel-based RPE sheets.

A current challenge to stem cell therapy involves the low purity of differentiated hiPSC-derived RPE cells. The pigment cells were manually separated from the nonpigment cells in this study, which may have resulted in cell waste and incomplete purification. The cell purification methods currently employed for the selection of specific cells include manual, enzymatic and flow cytometry-based sorting [82]. The development of a novel, simplified purification method is important for the clinical use of hPSC-differentiated cells in the future.

## Supplementary Material

rbaf035_Supplementary_Data

## Data Availability

Data will be made available up request.
