# Supplementary material for: Differentiation of human induced pluripotent stem cells into retinal pigment epithelium cells during culture on peptide-grafted hydrogels
Source: Regen Biomater. 2025 Apr 26;12:rbaf035. doi: 10.1093/rb/rbaf035 (PMC12098265; doi:10.1093/rb/rbaf035)
Supplement: rbaf035_Supplementary_Data [file rbaf035_supplementary_data.zip › 2 Supplementary N5.pdf]

Differentiation of human induced pluripotent stem cells into retinal pigment epithelium cells during culture on peptide-grafted hydrogels

Jun Liu[a,1], Qian Liu[a,1], Minmei Guo[a], Chengyu Jiang[a], Jianyang Cheng[a], Ting Wang[a], Tzu-Cheng Sung[a], Shih-Jie Chou[b,c], Shih-Hwa Chiou[b-d], Guoping Fan[e], Akon Higuchi[a,f,g,*]

[a]State Key Laboratory of Ophthalmology, Optometry and Visual Science, Eye Hospital, Wenzhou Medical University, No. 270, Xueyuan Road, Wenzhou, Zhejiang, 325027, China.

[b]Department of Medical Research, Taipei Veterans General Hospital, Taipei 112201, Taiwan

[c]Institute of Pharmacology, School of Medicine, National Yang Ming Chiao Tung University, Taipei 112304, Taiwan

[d]Department of Ophthalmology, Taipei Veterans General Hospital, Taipei 11217, Taiwan

[e]Department of Human Genetics, David Geffen School of Medicine, UCLA, Los Angeles, CA 90095 USA

[f]Department of Chemical and Materials Engineering, National Central University, No. 300, Jhongda RD., Jhongli, Taoyuan 32001, Taiwan

[g]R&D Center for Membrane Technology, Chung Yuan Christian University, Chungli, Taoyuan 320, Taiwan

* Correspondence author. State Key Laboratory of Ophthalmology, Optometry and Visual Science, Eye Hospital, Wenzhou Medical University, No. 270, Xueyuan Road, Wenzhou, Zhejiang, 325027, China & Department of Chemical and Materials Engineering, National Central University, No. 300, Jhongda RD., Jhongli, Taoyuan 32001, Taiwan China.

Tel.: +86 577-88068822; fax: +86 086-577-88832083.
E-mail address: higuchi@ncu.edu.tw; higuchi@wmu.edu.cn (A. Higuchi)

[1] These authors contributed equally to this work.

Total word count:8330

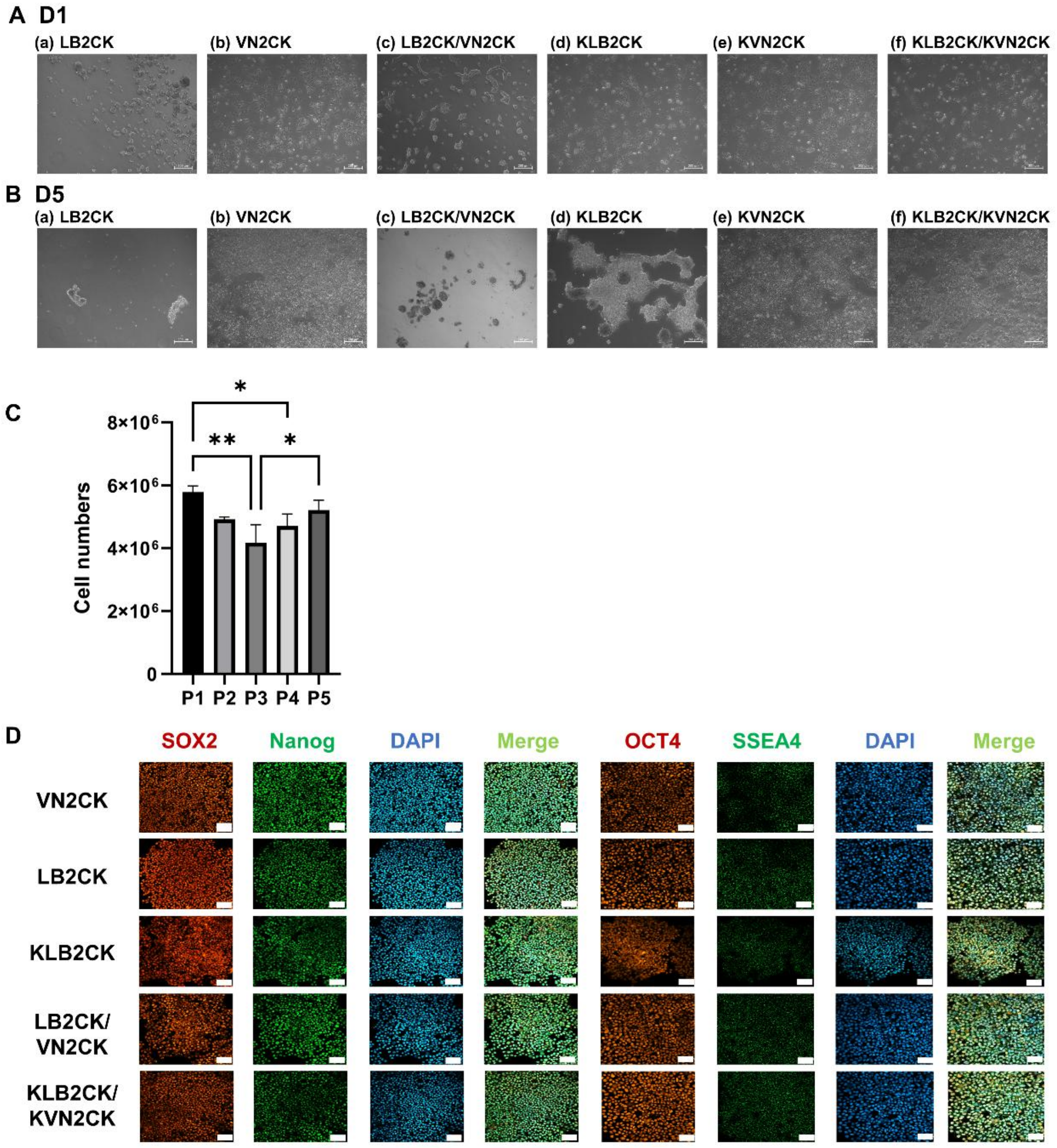

Supplementary Figure 1. hiPSCs (HPS0077) grown on the surface of several peptide-grafted PAI hydrogels. (A, B) Microscopy images of hiPSCs (HPS0077) grown on the surface of several peptide-grafted PAI hydrogels prepared with a peptide concentration of 1000 μg/mL at one day (A) and five days (B) after cell inoculation. Morphologies of hiPSCs on (a) LB2CK-, (b) VN2CK-, (c) LB2CK/VN2CK-, (d) KLB2CK-, (e) KVN2CK-, and (f) KLB2CK/KVN2CK-grafted PAI hydrogels. Scale bar: 200 μm. (C) Numbers of hiPSCs (HPS0077) grown on the surface of KVN2CK-grafted PAI hydrogels with a peptide concentration of 1000 μg/mL over 5 passages. (D) Immunohistochemical staining for the pluripotency biomarkers SOX2 (red), Nanog (green), Oct4 (red), and SSEA-4 (green) in hiPSCs cultured on VN2CK-, LB2CK-, KLB2CK-, LB2CKVN2CK-, KLBB2CK/KVN2CK-grafted PAI hydrogel surfaces with a peptide concentration of 1000 μg/mL. DAPI staining shows the cell nuclei (blue). Scale bar: 50 μm.

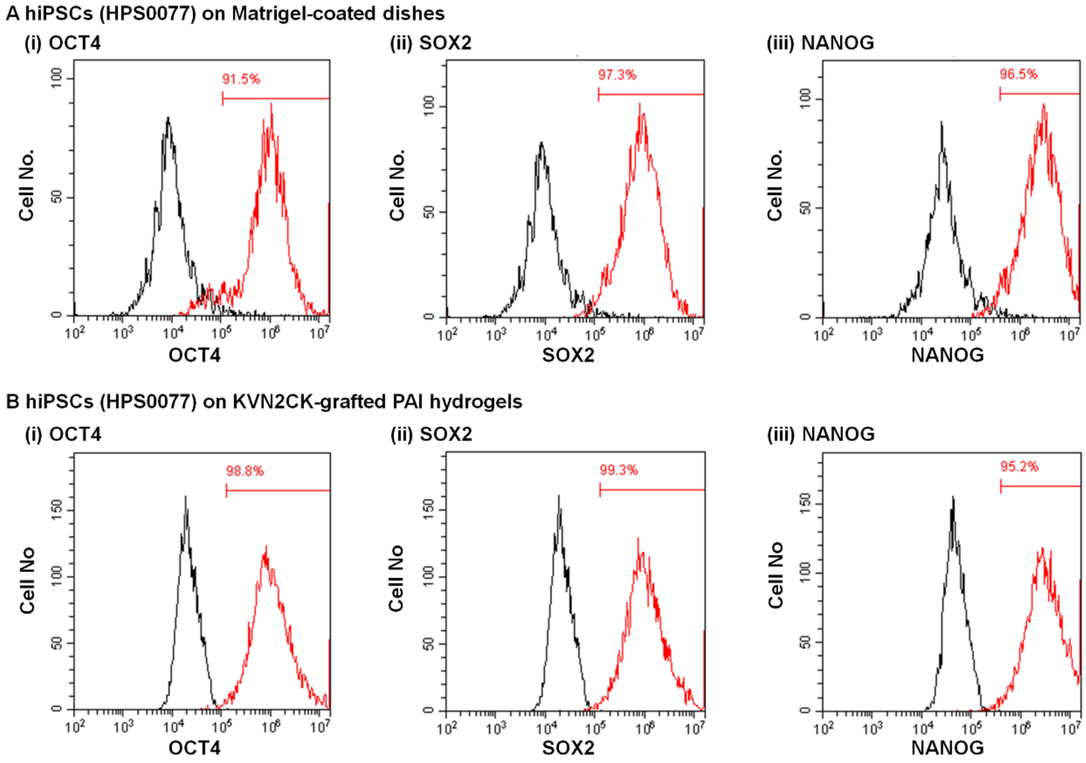

Supplementary Figure 2. Evaluation of the pluripotency capacity of hiPSCs (HPS0077) cultured on peptide-grafted PAI hydrogel surfaces. Flow cytometry histograms illustrating the expression of the pluripotency marker OCT4 (i), SOX2 (ii) and NANOG (iii) in hiPSCs cultured on the (A) Matrigel-coated surface and (B) KVN2CK-grafted PAI hydrogel surface prepared with a peptide concentration of 1000 μg/mL for five days.

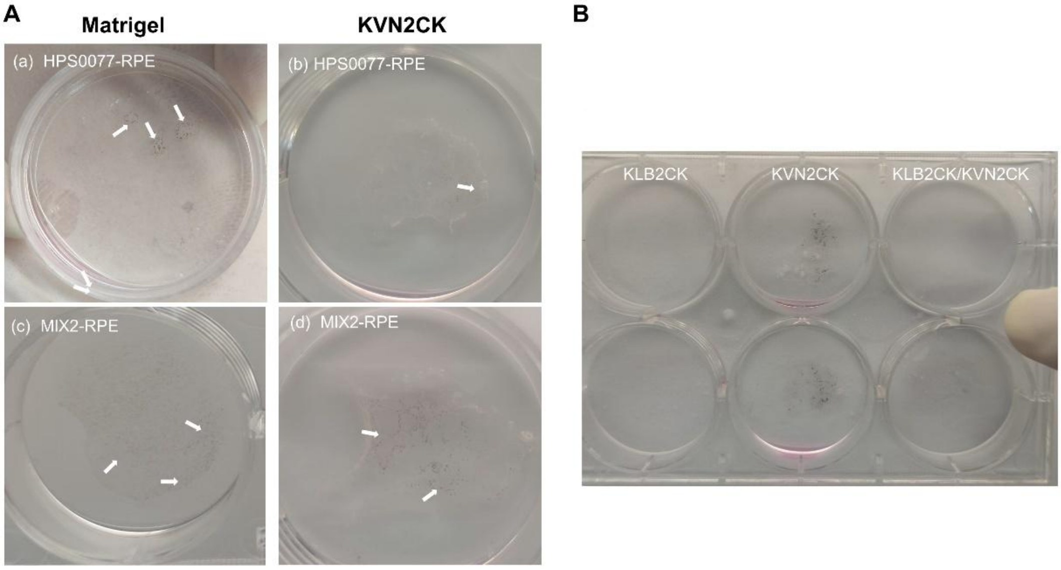

Supplementary Figure 3. **Differentiation of hiPSCs (HPS0077 and MIX2) into RPE cells via the NIC84 protocol.** (A) Image of pigmented cells produced after 42 days of differentiation. (a) Differentiation of hiPSCs (HPS0077 (a, b) and MIX2 (c, d)) into RPE cells during cultivation on a Matrigel-coated surface (a, c) and KVN2CK-grafted PAI hydrogels prepared with a peptide concentration of 1,000 µg/mL (b, d). (B) Photographs of hiPSC (HPS0077)-derived RPE cells cultivated on KLB2CK-grafted, KVN2CK-grafted, and KLB2CK/KVN2CK-grafted PAI hydrogel surfaces prepared with a peptide concentration of 1,000 µg/mL after 56 days of differentiation.

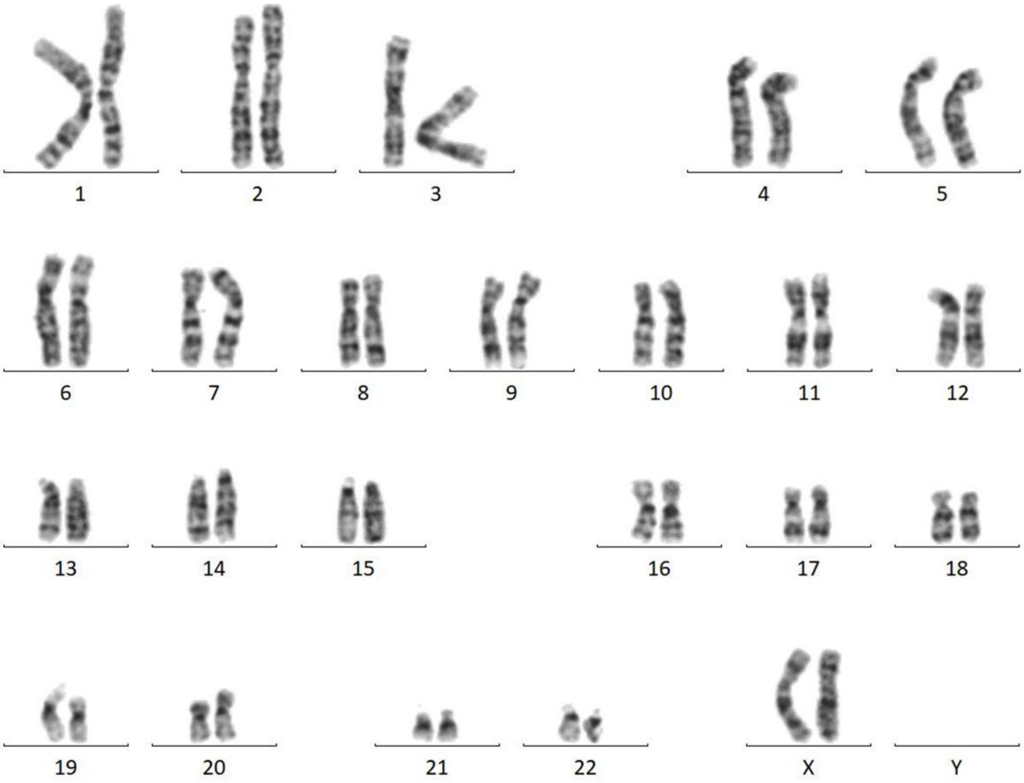

Supplementary Figure 4. Karyotype analysis of RPE cells derived from hiPSCs (MIX2) after 84 days of differentiation on the KVN2CK-grafted PAI hydrogel surface prepared at a peptide concentration of 1000 μg/mL.

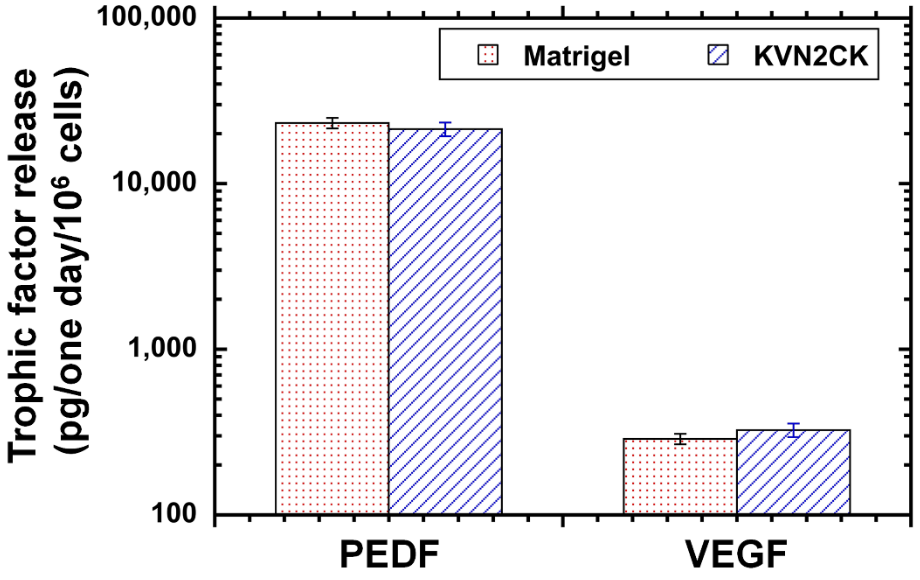

Supplementary Figure 5. Secretion of the trophic factors of PEDF and VEGF by hiPSC (HPS0077)-derived RPE cells on Matrigel-coated surface (left bar) and KVN2CK-grafted PAI hydrogel surface (right bar), expressed as the amount secreted per million cells after two day of culture, as detected by enzyme-linked immunosorbent assay (ELISA). PEDF: pigment epithelium-derived factor; VEGF: vascular endothelial growth factor.

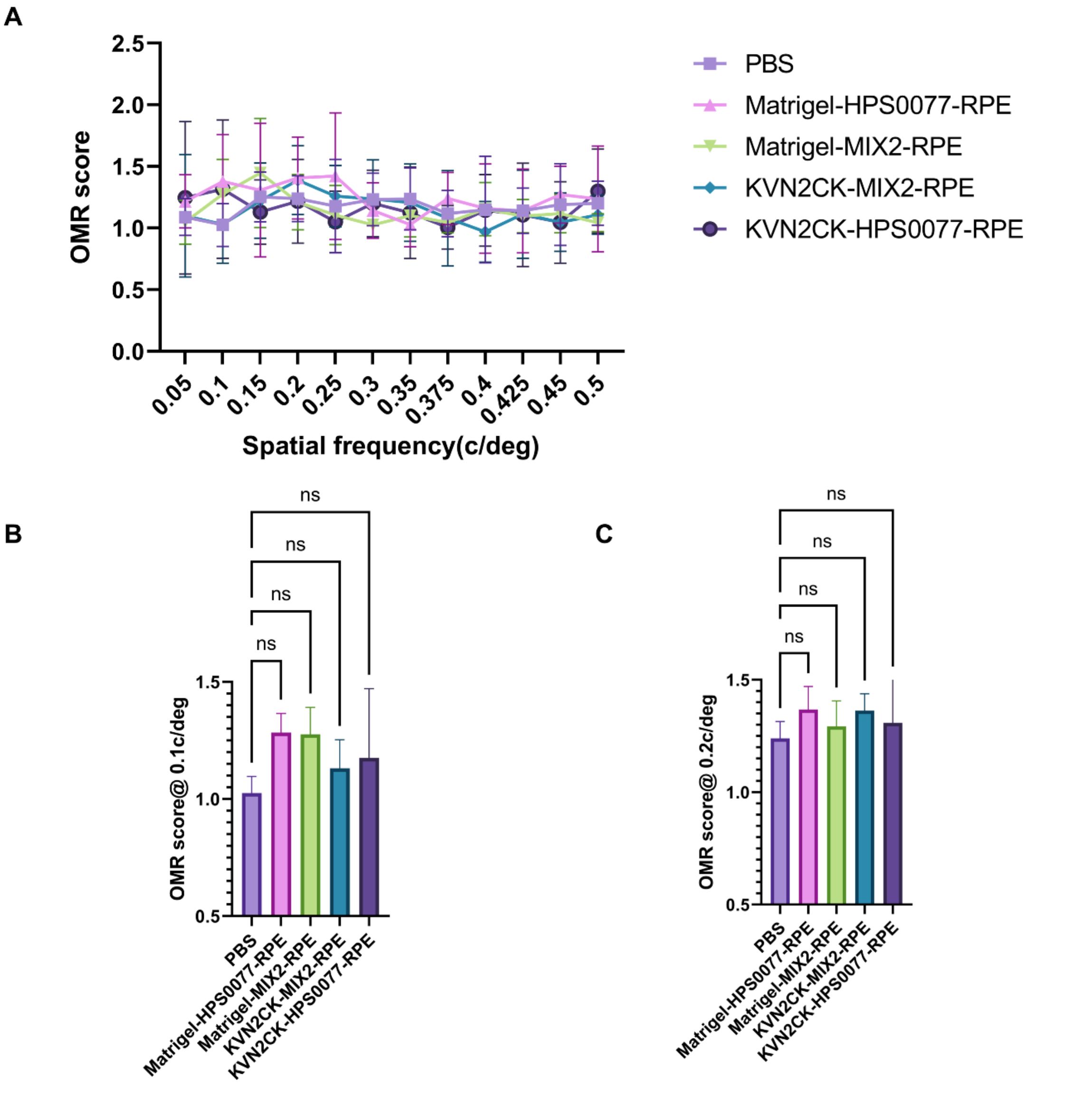

Supplementary Figure. 6 Evaluation of visual function in Royal College of Surgeons (RCS) rats following cell therapy. (A) OMR scores of RCS rats subjected to different stimuli after injection of PBS or RPE cells derived from HPS0077 and MIX2 hiPSCs cultured on KVN2CK-grafted PAI hydrogel surfaces prepared at a peptide concentration of 1000 μg/mL and Matrigel-coated surfaces four weeks after injection.

(B, C) OMR scores of RCS rats exposed to 0.1 c/deg (B) and 0.2 c/deg (C) stimuli after injection of PBS or RPE cells derived from HPS0077 and MIX2 hiPSCs cultured on the KVN2CK-grafted PAI hydrogel surface prepared at a peptide concentration of 1000 µg/mL and a Matrigel-coated surface four weeks after injection. "ns" indicates no statistically significant difference.

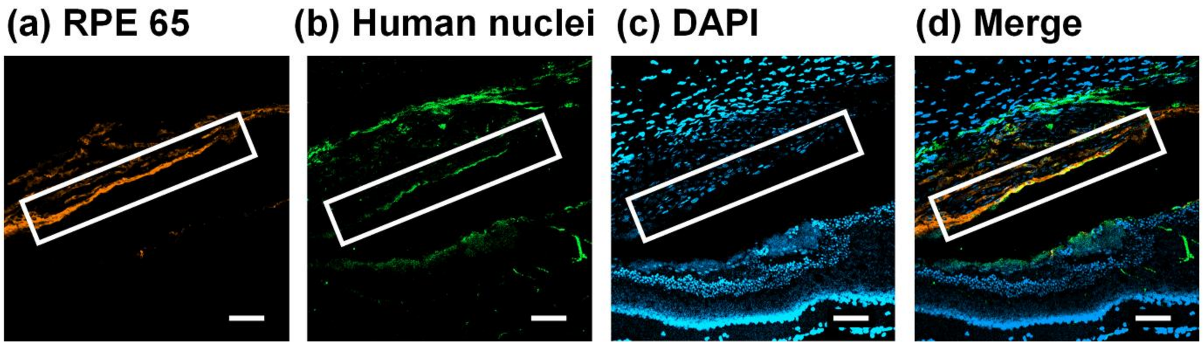

Supplementary Figure 7. Evaluation of the existence of hiPSC-derived RPE cells on retinas of RCS rats, which were transplanted subretinally. Representative immunohistochemical images of RPE65 (a, red) and human nuclei (b, green) as well as DAPI (c, blue) from retinas of RCS rats eight weeks after the transplantation of HPS0077-derived RPE cells, which were cultured on KVN2CK-grafted PAI hydrogel surfaces prepared at a peptide concentration of 1000 µg/mL. Photo (d) was created by merging (a) – (c). White frame area indicates double staining cells with RPE 65 and human nuclei antibodies as well as DAPI. Scale bar: 50 µm.
